# Identification of a novel gene signature related to prognosis and metastasis in gastric cancer

**DOI:** 10.1007/s13402-024-00932-y

**Published:** 2024-03-13

**Authors:** Joseba Elizazu, Aizpea Artetxe-Zurutuza, Maddalen Otaegi-Ugartemendia, Veronica Moncho-Amor, Manuel Moreno-Valladares, Ander Matheu, Estefania Carrasco-Garcia

**Affiliations:** 1 Cellular Oncology Group, Biodonostia Health Research Institute, Paseo Dr. Beguiristain s/n, San Sebastian, 20014 Spain; 2 CIBER de Fragilidad y Envejecimiento Saludable (CIBERfes), Madrid, 28029 Spain; 3 Pathology Department, Donostia University Hospital, San Sebastian, Spain; 4 IKERBASQUE, Basque Foundation for Science, Bilbao, 48009 Spain

**Keywords:** Gastric cancer, Prognosis, Biomarker signature, *ANKRD6*, EMT

## Abstract

**Background:**

Gastric Cancer (GC) presents poor outcome, which is consequence of the high incidence of recurrence and metastasis at early stages. GC patients presenting recurrent or metastatic disease display a median life expectancy of only 8 months. The mechanisms underlying GC progression remain poorly understood.

**Methods:**

We took advantage of public available GC datasets from TCGA using GEPIA, and identified the matched genes among the 100 genes most significantly associated with overall survival (OS) and disease free survival (DFS). Results were confirmed in ACRG cohort and in over 2000 GC cases obtained from several cohorts integrated using our own analysis pipeline. The Kaplan-Meier method and multivariate Cox regression analyses were used for prognostic significance and linear modelling and correlation analyses for association with clinic-pathological parameters and biological hallmarks. In vitro and in vivo functional studies were performed in GC cells with candidate genes and the related molecular pathways were studied by RNA sequencing.

**Results:**

High expression of *ANKRD6, ITIH3, SORCS3, NPY1R* and *CCDC178* individually and as a signature was associated with poor prognosis and recurrent disease in GC. Moreover, the expression of *ANKRD6* and *ITIH3* was significantly higher in metastasis and their levels associated to Epithelial to Mesenchymal Transition (EMT) and stemness markers. In line with this, RNAseq analysis revealed genes involved in EMT differentially expressed in *ANKRD6* silencing cells. Finally, *ANKRD6* silencing in GC metastatic cells showed impairment in GC tumorigenic and metastatic traits in vitro and in vivo.

**Conclusions:**

Our study identified a novel signature involved in GC malignancy and prognosis, and revealed a novel pro-metastatic role of *ANKRD6* in GC.

**Supplementary Information:**

The online version contains supplementary material available at 10.1007/s13402-024-00932-y.

## Introduction

Gastric cancer (GC) is the fifth most common cancer and the third leading cause of cancer death worldwide [1, 2]. Although the clinical management of GC has improved in the last decades and currently consists of surgical resection, chemoradiotherapy and targeted therapy [3], the prognosis is still dismal, with an average 5-year survival rate of around 20% [2]. This poor outcome is consequence of the high incidence of recurrence and metastasis. In this sense, around 30% of patients undergoing curative resection experiences recurrence within a year [4]. Moreover, a high proportion of patients (60%) undergoing resection display lymph node metastasis [5], even in early GC cases [6], whereas the 30–35% of the patients presents distant metastases spreading to the liver, peritoneum, lung, or bone at the time of diagnosis [7]. GC patients presenting recurrent or metastatic disease display a median life expectancy of only 8 months [8]. Altogether, this scenario points out the need of understanding the underlying molecular mechanisms of recurrence and metastasis in order to identify biomarkers for monitoring and suitable targets for prevention and inhibition.

GC is a heterogeneous disease influenced by different environmental factors and several genetic and epigenetic alterations. Traditionally, GC has been classified into intestinal and diffuse histological subtypes on the basis of the Lauren’s classification [9]. Over the past decade, advances in sequencing technology and high-throughput analysis delivered new insights and unravelled that GC presents high genetic and epigenetic molecular heterogeneity. Indeed, different consortiums, such as The Cancer Genome Atlas (TCGA) or the Asian Cancer Research Group (ACRG) carried out large scope studies, identifying critical driver genes and proposing molecular classifications of GC [10, 11 ]. Both consortiums identified four different molecular subtypes of GC and, in the case of the ACRG classification, the subtypes were associated with different survival rates and recurrence patterns [11]. These molecular studies helped to understand the biology of GC and represent very useful tools in basic research. However, although this knowledge exhibits potentially important clinical implications for disease diagnosis and treatment, the comprehension of its clinical connotations is a complex matter and these molecular classifications have not been translated in its current format into the clinic yet.

The main known risk factors for GC recurrence and metastasis are still related to the tumor stage, the extension of the surgery, the lymph node affectation, the tumor size and other non-molecular aspects [12–14], remaining the molecular determinants of GC progression unknown. In this scenario, we used the publicly available dataset of the GC patients from the TCGA cohort and identified the genes most significantly associated with GC prognosis. From this, and taking advantage of computational biology tools, additional patient datasets, clinical information and functional studies, we identified a novel gene signature related to recurrence and metastasis in GC, highlighting the special relevance of *ANKRD6* and *ITIH3,* which represent novel potential biomarkers for GC progression, EMT and metastasis, as well as suitable targets for the improvement of the management of the disease.

## Experimental procedures

### Patient cohorts

The GEPIA tool was used to identify the genes most associated with survival in GC patients (http://gepia.cancer-pku.cn/detail.php?clicktag=survival2). We have analyzed data from GC patients cohorts previously published by the TCGA, Cristescu et al. (ACRG cohort) [11], Oh SC et al. (MDACC, KUCM, KUGH and MDACC cohorts) [15], Ooi CH et al. (Singapore cohort) [16], Lee et al. (SMC cohort) [17], Cheong JH et al. (Yonsei cohort) [18], and Cho JY et al. (YUSH cohort) [19]. We downloaded the public raw expression data and clinical data from the Gene Expression Omnibus (GEO) repository.

### Processing of patient gene expression data

Processed Transcripts Per Million (TPM) expression data of the TCGA were downloaded with the TCGAbiolinks R package [20] prior to log2-normalization. Clinical data curated by the Genomic Data Commons (GDC) Data Portal were downloaded from Xena Browser [21]. Samples without OS data available were not included in the analyses.

All microarray data sets were pre-processed either by the Robust Multichip Average method for Affymetrix data with the oligo R package [22] or by quantile normalization for Illumina data before log2-transformation. Gene annotation data was obtained from BioMart, using the biomaRt R package [23], and for each data set and gene only the probe/bead that yielded the highest signal intensity value was used.

Microarray data were integrated first by sample-level standardization. Genes detected in at least 50% of the samples were kept, and missing expression data were imputed using nearest neighbour averaging with the R package impute. Those imputed values were subsequently eliminated after data set-derived batch effect removal using ComBat function of the sva R package. Standarized (Z-score) values were used in all patient analyses. Additionally, we downloaded log2(TPM+1) protein coding gene expression data from the DepMap Portal version 22Q2 [24].

### Cell culture

The gastric cancer cell line MKN45 was cultured as an adherent monolayer with some single round cells or clumps in suspension at 37 °C and 5% CO_2_ in RPMI medium (Gibco) supplemented with 10% FBS (Gibco), 100 U/ml penicillin and 100 µg/mL streptomycin. For experiments, the adherent monolayer cells were used. Cultures were regularly tested for *Mycoplasma*.

### *ANKRD6* silencing

For *ANKRD6* silencing by short hairpin RNA (shRNA), cells were infected with lentivirus harboring the TRCN0000158616 and TRCN0000162328 *shANKRD6* plasmids (Sigma, St. Louis, MO, USA) and the corresponding pLKO.1 puro control plasmid #8453 (*pLKO*) from Addgene, a gift from Dr. Bob Weinberg. Lentiviral infections were performed for 6 h and selected with puromycin.

### mRNA expression analysis

Total RNA was isolated using trizol (Life Technologies, Carlsbad, CA, USA). Reverse transcription was performed using the Maxima First Strand cDNA Synthesis Kit (ThermoFisher, Waltham, MA, USA) according to the manufacturer’s guidelines. Quantitative real-time PCR was performed in a CFX384 Thermal Cycler (Bio-Rad, Hercules, CA, USA) using Power SYBR^®^ Green Master Mix (ThermoFisher), 10 mmol/L of primers and 20 ng of cDNA. GAPDH was used as housekeeping gene. Statistical analysis was performed based on ΔCT values.

### Western blot

Protein lysates were resolved using sodium dodecyl sulfate-polyacrylamide gel electrophoresis and transferred to nitrocellulose membranes. The membranes were blocked and incubated overnight at 4 °C with primary antibodies against E-Cadherin (610181, BD Biosciences, San Jose, CA, USA), KRT18 (#4548, Cell Signaling Technology, Danvers, MA, USA) and β-actin (A5441, Sigma, St. Louis, MO, USA). Then, membranes were incubated with the corresponding secondary antibodies conjugated to horseradish peroxidase (HRP), and protein detection was performed using the NOVEX^®^ ECL system (Invitrogen, Carlsbad, CA, USA) and the iBright Imaging system (ThermoFisher).

### Immunofluorescence

Immunofluorescence was performed following standard procedures. Cells were seeded in 8-well immunofluorescence chamber slides (154534, LabTek ThermoFisher) and fixed with 4% PFA. Primary antibodies used were: cleaved-Caspase-3 (AF835, R&D Systems, Minneapolis, MN, USA), cleaved-PARP1 (ab32064, Abcam, Waltham, MA, USA), E-Cadherin (610181, BD Biosciences, San Jose, CA, USA) and KRT18 (#4548, Cell Signaling Technology). Secondary antibodies conjugated to fluorochromes were incubated and chromatin staining was performed with DAPI (D9542, Sigma). Cleaved-Caspase-3 and cleaved-PARP1 positive cells were counted manually and total number of cells were automatically quantified using the software for Bioimage Analysis QuPath v.0.4.3.

### Immunohistochemistry

Subcutaneous tumors were fixed with 3.7% formaldehyde and embedded in paraffin blocks that were cut into 5-µm-thick sections. Primary antibodies used were: E-Cadherin (610181, BD Biosciences, San Jose, CA, USA), active Caspase-3 (AF835, R&D Systems, Minneapolis, MN, USA) and Ki67 (ab15580, Abcam, Waltham, MA, USA). Sections were incubated with secondary antibodies and the stains were visualized using 3,3′-diaminobenzidine (DAB, ab64238, Abcam, Waltham, MA, USA). Nuclei were counterstained with hematoxylin. Images were captured with a ZEISS Axioscan 7 slide scanner, and image analyses were performed using Qupath v.0.4.3. All nuclei were detected automatically. E-Cadherin intensity means were obtained and averaged per tumor for statistical analysis, active Caspase-3 positive cells were quantified manually, and Ki67 staining was detected automatically by setting an optical density threshold.

### Proliferation

2·5×10^4^ cells were plated in duplicate in 6-well-treated plates. At days 1, 3 and 5 after seeding, cells were harvested using 0.05% trypsin-EDTA and counted using a Neubauer chamber and an inverted optical microscope.

### Migration and invasion

For migration and invasion transwell assays were used. Cells were starved for 24 h in serum-free medium. After that, migration was analyzed using Corning^®^ Transwell^®^ polycarbonate membrane inserts (#3422, Corning, NY, USA), and invasion was evaluated using the QCM™ Collagen Cell Invasion Assay of Millipore (ECM551, Burlington, MA, USA). Cells (10·10^4^ cells for migration and 25·10^4^ cells for invasion) were seeded into the upper compartment in medium without serum, and the inserts were placed in wells of 24-well flat bottom plates containing medium with 10% of a fetal bovine serum as chemo-attractant. After 96 h, the migrated and invading cells were fixed and stained according to the manufacturer’s protocol. Cells on the upper side of the chambers were wiped off using cotton swaps, and for quantification, the staining of cells situated in the undersurface of the membranes was dissolved and the absorbance was measured at 560 nm.

### Cell cycle analysis by flow cytometry

The experiment was carried out immediately after antibiotic selection was complete. Cells were harvested using trypsin, washed with PBS and counted using a Neubauer chamber. 1·10^6^ cells were fixed with 75% cold ethanol at −20 °C for at least 1 h, and incubated with 0.5% Triton X-100 and 25 µg/mL RNase A in PBS for 30 min at room temperature. Then, DNA was stained with 25 ng/mL propidium iodide for 15 min and samples were analyzed using a CytoFLEX flow cytometer (Beckman Coulter Co., Miami, FL, USA).

### In vivo carcinogenesis assays

For subcutaneous xenografts, MKN45 cells were harvested with trypsin/EDTA and resuspended in PBS. 1 × 10^5^ cells were injected subcutaneously into both flanks of Foxn1^nu^/Foxn1^nu^ nude mice (8 weeks old). An external calliper was used to measure tumor size at the indicated time points, from which tumor volume was calculated according to the formula ½ (*length* × *width*^2^). Wilcoxon rank sum test was used to test the dimensions of the tumors independently for each day, and the global impact of *ANKRD6* silencing with either of the *shANKRD6* plasmids was assessed by a longitudinal linear mixed model.

### RNA sequencing

Total RNA from *pLKO* and *shANKRD6* MKN45 cells was isolated using trizol. For library preparation, the DNBSEQ Eukaryotic Stranded Transcriptome library preparation pipeline was followed. Sequencing data filtering was performed using the SOAPnuke software [25], developed by BGI. Data were aligned to the GRCh38.p13 reference genome using Bowtie2.

### Functional enrichment analysis

The Gene Set Variation Analysis (GSVA) method [26] was used to assess the prognostic value of the gene signatures evaluated. The GSVA method was also applied to estimate the relative enrichment of the hallmark gene sets from the Molecular Signature Database [27] in the TCGA and ACRG cohorts. Differential enrichment analysis (DEA) was performed using DESeq2 [28], adjusting for the first principal component to remove unwanted variation and using the likelihood ratio test. Then hallmark Gene Set Enrichment Analysis was performed with the R package clusterProfiler [29].

### Statistical analysis

The top-100 genes most correlated with OS and DFS in GC were identified using GEPIA (http://gepia.cancer-pku.cn/index.html) [30]. The prognostic value of *ANKRD6, ITIH3, SORCS3, NPY1R* and *CCDC178* expression in GC was explored in the GC cohorts through Kaplan-Meier and multivariate Cox regression analyses. In the former, we used the best cut-off method implemented in the package survminer (https://cran.r-project.org/web/packages/survminer/index.html) to distribute the samples into high and low expression and GSVA score groups. In the latter, we used the uncut values in a model adjusted for age and TNM stage. The prognostic value of *ANKRD6, ITIH3, SORCS3, NPY1R* and *CCDC178* expression in the different cancer types from the TCGA was assessed in a Cox univariate regression independently for each cohort without expression level grouping.

We used parametric (*T*-test or pairwise *T*-test *p*-value adjustment for variables with more than 2 groups) and non-parametric (Wilcoxon rank sum test or Dunnett’s test) tests to assess the relevance of the five genes in relation to the different clinic-pathological characteristics. We also used a paired *T*-test to analyse the differences in expression between GC tissue and normal gastric tissue in the ACRG cohort (N = 100).

The correlation between the expression of *ANKRD6, ITIH3, SORCS3, NPY1R* and *CCDC178*, and the correlation of each with the expression of epithelial and mesenchymal markers, EMT inductors, stem cell regulators and hallmark enrichment scores were determined using the Spearman’s rank correlation test in GC tissue from patients of the TCGA and the ACRG cohorts. Correlation coefficients were grouped by hierarchical clustering based on Euclidean distance.

The *p* values were adjusted with the Benjamini-Hoichberg (BH) method when necessary, and statistical significance was considered when *p* < 0.05 (*p < 0.05, **p < 0.01, ***p < 0.001). All statistical analyses were conducted using R Statistical Software (v4.3.1; R Core Team 2023).

## Results

### Six transcripts match among the 100 most correlated with OS and DFS in GC

**Fig. 1:**
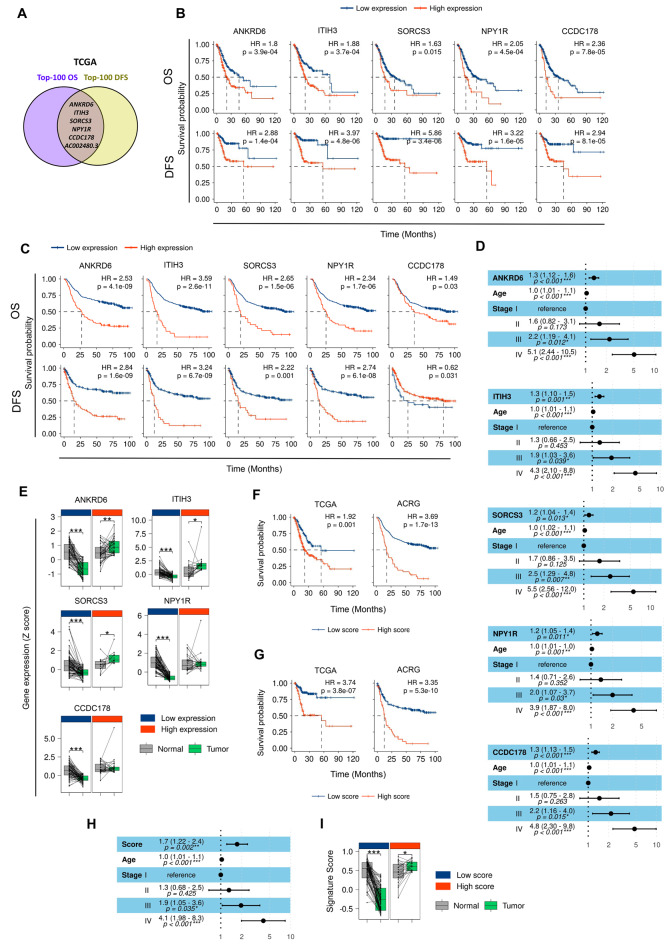
Prognostic potential of *ANKRD6, ITIH3, SORCS3, NPY1R* and *CCDC178* in GC. **A** *ANKRD6, ITIH3, SORCS3, NPY1R, CCDC178* and the lncRNA *AC002480* are the common transcripts among the top-100 transcripts most significantly associated with OS and DFS in the GC TCGA cohort (STAD dataset analyzed in GEPIA). Kaplan-Meier plots of OS and DFS according to the expression of the 5 cited coding genes in the TCGA (**B**) and ACRG (**C**) cohorts using the best cut-off method for expression level stratification. **D** Forest plots showing multivariate Cox regression analysis for *ANKRD6, ITIH3, SORCS3, NPY1R* and *CCDC178* expression in association with OS in the TCGA cohort, adjusting for age and TNM stage. **E** Expression of the identified genes in normal gastric tissue versus the corresponding paired GC tissue in ACRG patients stratified into high and low expression subgroups according to the cut-offs used in the OS analysis from C (N = 100). Kaplan-Meier plots of OS (**F**) and DFS (**G**) according to the score of the 5-gene GSVA signature composed by *ANKRD6, ITIH3, SORCS3, NPY1R* and *CCDC178* in the TCGA (left) and ACRG (right) cohorts using the best cut-off method for GSVA score stratification. **H** Forest plot showing multivariate Cox regression analysis for the 5-gene GSVA score in association with OS in the TCGA cohort, adjusting for age and TNM stage (**I**). 5-gene GSVA score in normal gastric tissue versus the corresponding paired GC tissue in ACRG patients stratified into high and low score subgroups defined in the OS analysis from F (right) (N = 100)

Using the GEPIA tool, we identified the top-100 transcripts most significantly associated with overall survival (OS) and disease-free survival (DFS) in the GC patients from the TCGA (n = 408) (Supplementary file 1). Among them, 6 were associated with both OS and DFS (Fig. 1A). These include the coding genes *ANKRD6* (Ankyrin Repeat Domain 6), *ITIH3* (inter-alpha-trypsin inhibitor heavy chain 3)*, SORCS3* (sortilin related VPS10 domain containing receptor 3)*, NPY1R* (neuropeptide Y receptor Y1), *CCDC178* (coiled-coil domain containing 178), and the long non-coding RNA *AC002480.3*. In particular, we focused on the 5 genes and we confirmed that their high individual expression was associated with reduced OS and DFS in the TCGA (Fig. 1B), results that were extended to the ACRG cohort (n = 299) (Fig. 1C). To further characterize their impact on survival, we performed a multivariate Cox proportional hazards regression model, adjusting for relevant factors including age and TNM tumor stage. This analysis revealed that the expression of the 5 genes represented an increased risk for OS in the TCGA patients (Fig. 1D), and these results were confirmed for *ANKRD6, ITIH3, SORCS3* and *NPY1R* in the ACRG cohort (Fig. S1A). In relation to DFS, *ANKRD6, ITIH3, NPY1R* and *CCDC178* represented also an increased risk for DFS in TCGA (Fig. S1B), and *ANKRD6, ITIH3* and *NPY1R* in ACRG (Fig. S1C).

We next compared their expression in tumor and normal gastric samples. For this, we distributed the cases between patients with high or low tumor expression of the genes according to the cut-offs used for survival. For the 5 genes, the expression in normal tissue was similar between the groups of low or high survival, but the trend observed in the tumor samples was different between these groups. Noteworthy, overexpression of *ANKRD6, ITIH3* and *SORCS3* was detected in tumor compared with control tissue in the patients presenting poor survival (Fig. 1E). On the contrary, cases with extended survival displayed lower levels of the 5 genes in the tumor compared to healthy tissue (Fig. 1E).

We wondered whether the identified genes could represent a malignant signature associated to cancer and studied their prognostic potential in additional cancer types. For this, we took advantage of publicly available datasets of TCGA studies, and analyzed 32 cancer types. This analysis revealed interesting results, as some of the genes were associated with poor prognosis in several cancer types (Fig. S2). For instance, the expression of *ANKRD6* and *ITIH3* was associated with reduced OS in colon carcinoma (COAD) and kidney renal papillary cell carcinoma (KIRP), and the expression of *CCDC178* in bladder urothelial carcinoma (BLCA) and thyroid carcinoma (THCA).

Next, we performed a Gene Set Variation Analysis (GSVA) to obtain a global score corresponding to the combined expression of the 5 genes in GC. We explored the potential of the score to predict prognosis in both TCGA and ACRG cohorts, and using Kaplan-Meier estimator, we identified that the high score of the signature was associated to reduced OS (Fig. 1F) and DFS (Fig. 1G). Besides, multivariate Cox regression analyses showed that the score of the signature constituted an independent prognostic factor for OS after adjusting for the risk factors age and tumor stage in both cohorts. Notably, the Hazard Ratios (HRs) of the signature were superior to those of the 5 genes individually (Figs. 1H and S1D). Similar results were obtained with DFS (Fig. S1E, F). Moreover, when we analyzed paired tumor *versus* gastric healthy tissue samples, the signature score was significantly higher in those subsets of patients presenting poor outcome (Fig. 1I). Concerning the other cancer types, there was not a general pattern linking the expression of the 5 genes to survival in additional cancer types (Fig. S2). These results reveal that the 5 identified genes individually or as a signature serve as age- and tumor stage- independent prognostic factors in GC.

### High expression of the identified genes correlated with tumor recurrence and metastasis in GC

**Fig. 2:**
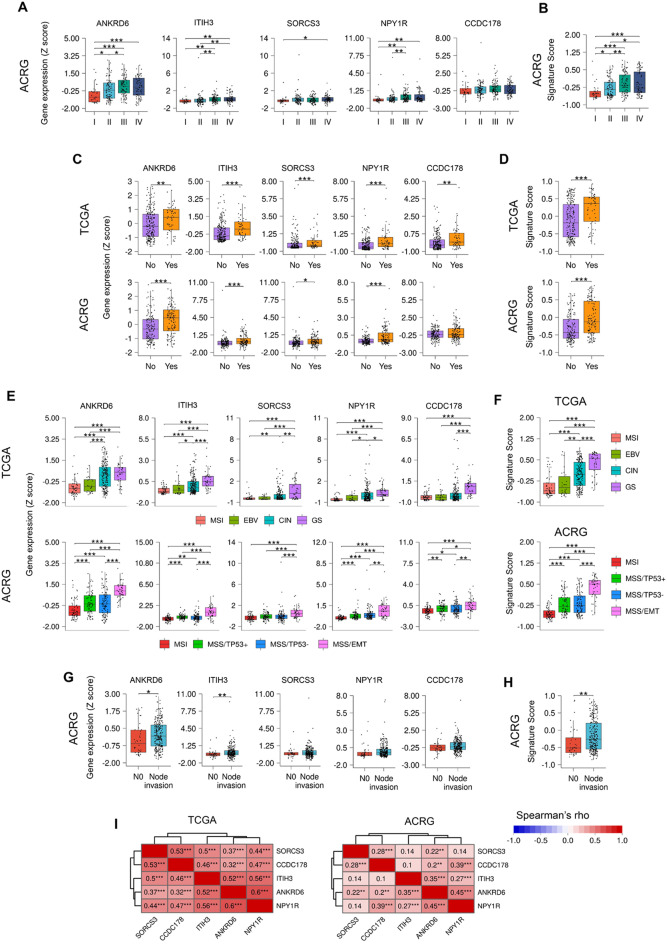
Association of *ANKRD6, ITIH3, SORCS3, NPY1R* and *CCDC178* expression with clinic-pathological characteristics in TCGA and ACRG GC cohorts. Expression of the 5 genes individually (**A**) and as a GSVA signature (**B**) in the different tumor stages in the ACRG cohort (N_I_ = 30, N_II_ = 96, N_III_ = 95, N_IV_ = 77). Expression of the 5 genes individually (**C**) and as a GSVA signature (**D**) in primary tumor tissue of patients who recurred (Yes) or not (No) after the primary therapy in the TCGA and ACRG cohorts (TCGA: N_No_ = 216, N_Yes_ = 58; ACRG: N_No_ = 157, N_Yes_ = 125). Expression of the 5 genes individually (**E**) and as a GSVA signature (**F**) in the different molecular subtypes of GC identified by the TCGA (top) and the ACRG (bottom). *TCGA classification*: MSI: GC with microsatellite instability (N = 55); EBV: Epstein-Barr virus-positive GC (N = 26) CIN: GC with chromosomal instability (N = 195), and GS: genomically stable GC (N = 42). *ACRG classification*: MSI: GC with microsatellite instability (N = 68); MSS/TP53+: microsatellite-stable with active TP53 (N = 79), MSS/TP53-: microsatellite-stable with inactive TP53 (N = 107); and EMT: microsatellite stable with epithelial-to-mesenchymal transition phenotype (N = 46). Expression of the 5 genes individually (**G**) and as a GSVA signature (**H**) in primary tumor tissue of patients presenting nodal dissemination (Node invasion) or not (N0) in ACRG. Node invasion group includes samples from N1 to N4 (N_N0_ = 38, N_Node invasion_ = 262). **I** Spearman correlation between *ANKRD6, ITIH3, SORCS3, NPY1R* and *CCDC178* in TCGA (left) and ACRG (right). Genes are grouped by hierarchical clustering. Red colour represents positive correlations (Colour figure online)

Next, we analyzed the expression of the identified genes in the different TNM stages of the disease. Overall, the expression of the 5 genes individually increased gradually according to the stage, being the differences in expression among stages significant for *ANKRD6, ITIH3* and *NPY1R* (Figs. 2A and S3A). Similarly, the signature also displayed significant differences (Figs. 2B and S3B). We also studied the expression of the genes in relation to recurrence and found that the expression of all of them individually or as a signature was significantly higher in the biopsies of those patients that underwent recurrence (Fig. 2C, D). Then, we explored their expression in the different molecular subtypes of GC proposed by the TCGA and the ACRG. This study unveiled that for all the genes, the MSI subtype presented the lowest expression in both cohorts (Fig. 2E). Of note, this subtype displayed the best prognosis in the ACRG classification, further indicating the link of the 5 genes with GC malignancy. Consistent with this idea, the highest expression of the 5 genes in the ACRG was registered in the MSS/EMT subtype, that with the poorest outcome and more prone to recur [10]. In the TCGA, the highest expression was detected in the genomically stable (GS) subtype (Fig. 2E), the one which is classified principally as MSS/EMT when the ACRG criteria are applied to the TCGA patients [10]. These results were reproduced by the 5-gene signature (Fig. 2F) and suggest a link with the process of EMT.

To characterize a potential interaction with metastasis, we investigated their link with lymph node dissemination and ascertained that the expression of the identified genes was higher in the patients of the ACRG exhibiting lymph node affectation, being the difference significant for *ANKRD6* and *ITIH3* (Fig. 2G). In the TCGA cohort, the differences, although less remarkable, confirmed the higher expression of *ANKRD6* when the nodes are invaded (Fig. S3C). For the 5-gene signature we found a significantly higher expression in samples from patients exhibiting positive nodes in the ACRG cohort (Figs. 2H and S3D).

To further explore the association between the identified genes, we analyzed the Spearman correlation between the 5 genes in the TCGA and ACRG cohorts, finding a positive correlation for all the pairs of genes in both of them (Fig. 2I). Moreover, the highest correlations were observed between *ANKRD6* with *ITIH3* and *NPY1R* (Fig. 2I). Overall, these results reveal an association of the identified genes with metastasis, and postulate *ANKRD6* and *ITIH3* as the strongest genes within the signature.

### Integrated analysis with data from 2000 GC patients across 9 independent cohorts confirms association with metastasis and recurrence

**Fig. 3:**
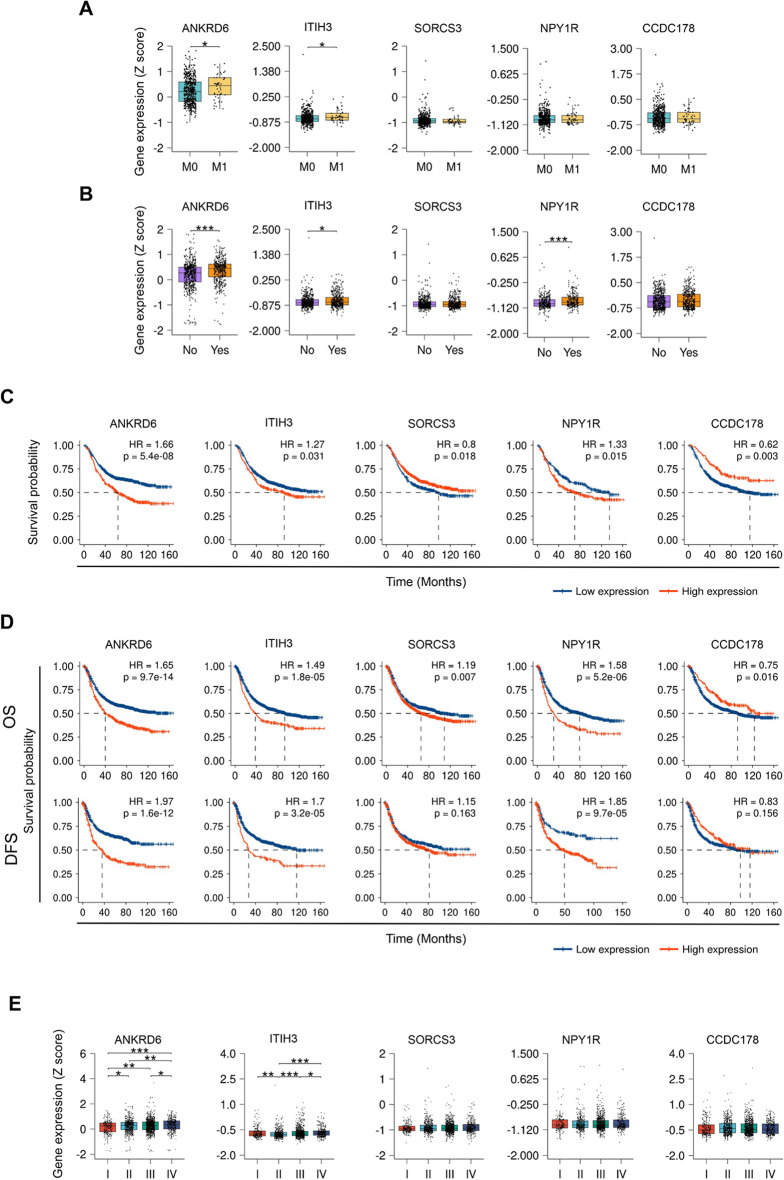
Association of *ANKRD6, ITIH3, SORCS3, NPY1R* and *CCDC178* expression with metastasis, recurrence and survival on a total of 1908 GC patients from 9 publicly available GC data sets. **A** Expression of the 5 genes in primary tumor tissue of GC patients presenting distant metastasis (M) or not (M0) (N_M0_ = 513, N_M1_ = 45). **B** Expression of the 5 genes individually in primary tumor tissue of patients who recurred (Yes) or not (No) after the primary therapy (N_No_ = 573, N_Yes_ = 423). **C** Kaplan-Meier plots of OS according to the expression of the 5 genes in patients treated with adjuvant chemotherapy (N = 1036). The best cut-off method was used for expression level stratification. **D** Kaplan-Meier plots of OS (top) and DFS (bottom) according to the expression of the 5 genes. The best cut-off method was used for expression level stratification. **E** Expression of the 5 genes in the different tumor stages (N_I_ = 217, N_II_ = 495, N_III_ = 879, N_IV_ = 316)

Next, with the aim of extending the study of the 5 genes in GC metastasis, we developed an analysis pipeline and we jointly analyzed available datasets corresponding to a total of 1908 GC patients from 9 independent cohorts (Table 1). We assessed the expression of the 5 genes in relation to metastasis, detecting significantly higher expression of *ANKRD6* and *ITIH3* in the samples belonging to patients suffering from metastatic disease (Fig. 3A). Furthermore, higher expression of *ANKRD6, ITIH3* and *NPY1R* was also detected in the samples of the patients exhibiting recurrent disease (Fig. 3B). Since recurrence and therapy resistance are related processes, we explored the association of the genes with the survival specifically in treated patients, revealing reduced OS in treated patients with high *ANKRD6, ITIH3* and *NPY1R* expression (Fig. 3C), and being the expression of *ANKRD6* and *NPY1R* independent prognostic factors in treated patients (Fig. S4). We also studied in this large set of patients the association with survival and tumor stage to confirm the data obtained in the TCGA and ACRG cohorts. Notably, high levels of *ANKRD6, ITIH3* and *NPY1R* significantly correlated with reduced OS, whilst high expression of *ANKRD6, ITIH3, SORCS3* and *NPY1R* was associated with lower DFS (Fig. 3D). Moreover, the multivariate Cox regression analysis showed that *ANKRD6, ITIH3* and *NPY1R* constituted independent prognostic factors for OS (Fig. S5) and DFS (Fig. S6). In relation to expression, we observed a progressive and significant increase of *ANKRD6* and *ITIH3* across the tumor stages (Fig. 3E). These results highlight the usefulness of the pipeline, validate the results obtained in TCGA and ACRG, and denote the clinical impact of the identified genes, with special emphasis for *ANKRD6* and *ITIH3* in GC malignancy, recurrence and metastasis.

**Table 1:** Cohorts characteristics

	ACRG (N = 299)	China (N = 126)	KUCM (N = 109)	KUGH (N = 93)	MDACC (N = 40)	Singapore (N = 182)	SMC (N = 432)	Yonsei (N = 564)	YUSH (N = 63)
GEO accession	GSE62254	GSE29272	GSE26901	GSE26899	GSE28541	GSE15459	GSE26253	GSE183136	GSE13861
Sex, n (%)									
Male	199 (66.6%)	99 (78.6%)	69 (63.3%)	73 (78.5%)	27 (67.5%)	117 (64.3%)	280 (64.8%)	384 (68.1%)	46 (73%)
Female	100 (33.4%)	27 (21.4%)	40 (36.47%)	20 (21.5%)	13 (32.5%)	65 (35.7%)	152 (35.2%)	180 (31.9%)	12 (27%)
Age, median (range)	64 yr (24–86 yr)	59 yr (23–71 yr)	58 yr (28–74 yr)	60 yr (36–83 yr)	58 yr (33–78 yr)	66 yr (23–92 yr)	52 yr (23–74 yr)	61 yr (27–86 yr)	62 yr (31–83)
DFS data available, n(%)	299 (100%)	No	109 (100%)	93 (100%)	No	No	432 (100%)	No	63 (100%)
M stage, n (%)									
M0	272 (91%)		102 (93.6%)	85 (91.4%)					54 (85.7%)
M1	27 (9%)		7 (6.4%)	7 (7.5%)					4 (6.3%)
Nodal stage, n (%)									
N0	38 (12.7%)	26 (20.6%)						292 (51.8%)	
N1-4	261 (87.3%)	98 (77.8%)						267 (47.3%)	
Tumor stage, n (%)									
I	30 (10%)	5 (4%)	40 (36.7%)	11 (11.8%)	1 (2.5%)	30 (16.5%)	68 (15.7%)	20 (3.5%)	12 (19%)
II	97 (32.4%)	5 (4%)	18 (16.5%)	18 (19.4%)	6 (15%)	27 (14.8%)	167 (38.7%)	145 (25.7%)	12 (19%)
III	96 (32.1%)	108 (85.7%)	36 (33%)	27 (29%)	12 (30%)	67 (36.8%)	130 (30.1%)	379 (67.2%)	24 (38.1%)
IV	76 (25.4%)	8 (6.3%)	15 (13.8%)	36 (38.7%)	21 (52.5%)	58 (31.9%)	67 (15.5%)	20 (3.5%)	15 (23.8%)
Lauren classification, n (%)									
Intestinal	146 (48.8%)		82 (75.2%)	59 (63.4%)		93 (51.1%)	139 (32.2%)	194 (34.4%)	19 (30.2%)
Diffuse	134 (44.8%)		11 (10.1%)	31 (33.3%)		12 (39.6%)	280 (64.8%)	196 (34.8%)	28 (44.4%)
Mixed	17 (5.7%)		5 (4.6%)	2 (2.2%)		17 (9.3%)	13 (3%)	25 (4.4%)	12 (19%)
Other	2 (0.7%)							148 (26.2%)	
Adjuvant chemotherapy, n (%)									
No			70 (64.2%)	26 (28%)			432 (100%)	109 (19.3%)	16 (25.4%)
Yes			39 (35.8%)	67 (72%)				451 (80%)	47 (74%)

### *ANKRD6* and *ITIH3* in GC progression, metastasis and EMT

**Fig. 4:**
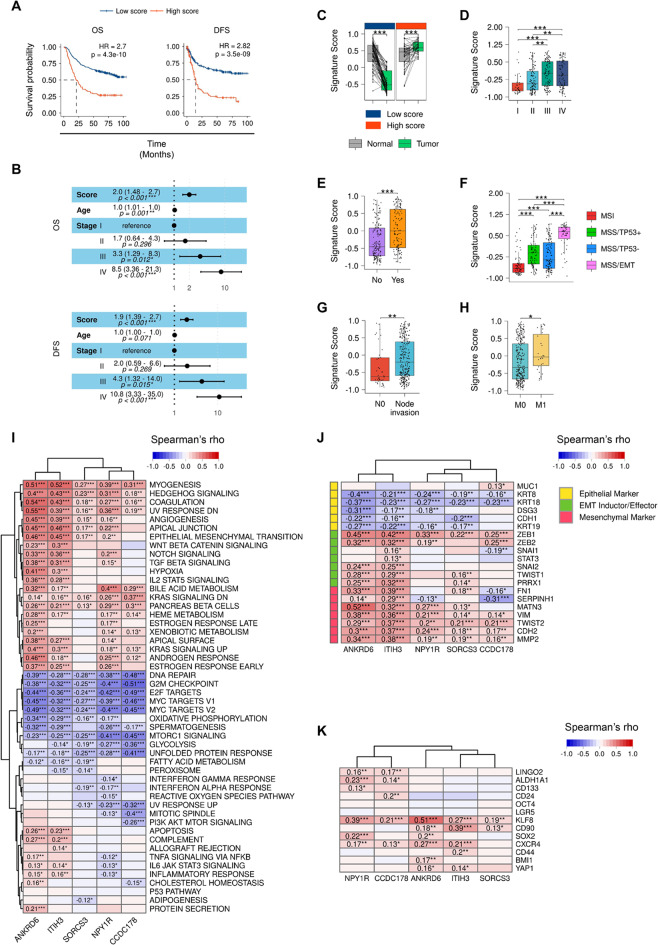
Association of the GSVA score of *ANKRD6* and *ITIH3* with prognosis and clinic-pathological characteristics, and correlation of the 5 genes with hallmark gene sets in the ACRG cohort. **A** Kaplan-Meier plots of OS and DFS according to the *ANKRD6* and *ITIH3* GSVA score in the ACRG cohort using the best cut-off method for GSVA score stratification. **B** Forest plots showing multivariate Cox regression analysis for the *ANKRD6* and *ITIH3* GSVA score in association with OS (left) and DFS (right), adjusting for age and TNM stage, in the and ACRG cohort. **C** *ANKRD6* and *ITIH3* GSVA score in normal gastric tissue versus the corresponding paired GC tissue in ACRG patients stratified into high and low score subgroups according to the cut-off defined in (**A**). *ANKRD6* and *ITIH3* GSVA score according to tumor stage (**D**), recurrence (**E**), molecular subtype (**F**), N stage (**G**), and M stage (**H**). **I** Correlation of *ANKRD6, ITIH3, SORCS3, NPY1R* and *CCDC178* with the GSVA scores of the 50 hallmark gene sets defining biological states or processes from The Molecular Signatures Database (MSigDB) in the ACRG cohort. Genes and hallmarks are grouped by hierarchical clustering. Blue colour represents negative correlations and red colour positive correlations. Spearman’s rho correlation coefficient is indicated in significant correlations. Correlation of *ANKRD6, ITIH3, SORCS3, NPY1R* and *CCDC178* with epithelial and mesenchymal markers, and EMT inductors (**J**), and with CSC markers (**K**) in the ACRG cohort. In (**J**), markers are clustered within each marker category, and in (**K**), genes in columns and rows are grouped by hierarchical clustering. Blue colour represents negative correlations and red colour positive correlations. Spearman’s rho correlation coefficient is indicated in significant correlations (Colour figure online)

Given the higher clinical impact of *ANKRD6* and *ITIH3,* we explored the score of their combined expression and compared its potential with respect to the 5 genes individually and the 5-gene signature. First, we detected that a high score of this 2-gene signature in ACRG and TCGA cohorts predicted worse prognosis (Figs. 4A and S7A), and was an age- and stage- independent prognostic factor (Figs. 4B and S7B), in the former cohort with HRs higher than those obtained with the genes individually. The 2-gene score was significantly higher in tumor *versus* paired normal gastric tissue in patients presenting poor outcome (Fig. 4C), and was also higher in advanced disease stages (Figs. 4D and S7C) and in patients with recurrence (Figs. 4E and S7D). Moreover, the score was higher in the MSS/EMT and GS molecular subtypes (Figs. 4F and S7E), in samples from patients presenting node invasion (Figs. 4G and S7F), and in metastatic cases in TCGA and ACRG (Figs. 4H and S7G). In the metastatic patients, the score of the 5-gene signature was not significantly higher neither in the TCGA cohort nor in the ACRG cohort (Fig. S8A), reinforcing the relevance of *ANKRD6* and *ITIH3* in metastasis. Moreover, using the Cancer Cell Line Encyclopedia (CCLE), we determined that among our identified genes, in GC cells, *ANKRD6* showed the highest levels on average, with the metastatic cell lines displaying higher expression than those cells derived from primary cases (Fig. S8B, S8C).

Then, with the aim of exploring the biological significance of the identified genes in GC, we studied their relationship with described pathways. For this, we took advantage of the GSVA signature score of the hallmark gene sets included in MsigDb (https://www.gsea-msigdb.org/gsea/msigdb/). The Spearman correlation analysis showed that the 5 genes presented positive correlation with the hallmarks of Epithelial mesenchymal transition (EMT), KRAS, Hedgehog signaling, or myogenesis in both TCGA and ACRG cohorts (Figs. 4I and S7H). In contrast, there was negative correlation with G2M checkpoint or DNA repair hallmarks (Figs. 4I and S7H). These analyses also showed differences in the pattern of correlations of the genes, differentiating *ANKRD6* and *ITIH3* in one side, from *SORCS3, NPY1R* and *CCDC178*. Thus, TGF beta, STAT3 and TNFA signaling and apoptosis were pathways specifically correlated with *ANKRD6* and *ITIH3* (Figs. 4I and S7H). Moreover, *ANKRD6* and *ITIH3* were the genes most positively correlated with the hallmark of EMT in both cohorts, with Spearman coefficients of 0.46 and 0.35 for *ANKRD6* in ACRG and TCGA; and coefficients of 0.44 and 0.49 for *ITIH3* in the corresponding cohorts (*p*-value < 0.001 in all cases) (Figs. 4I and S7H).

To further characterize the connection of these genes with the process of EMT, we explored the correlations established between them and canonical epithelial and mesenchymal markers, whose expression decrease and increase in cells undergoing EMT respectively, as well as with EMT inducers. This study revealed negative correlations with epithelial genes, such as the epithelial cell adhesion protein E-Cadherin (*CDH1*), the cytoskeletal genes coding the keratins 8, 18 and 19 (*KRT8, KRT18* and *KRT19)* and desmoglein 3 (*DSG3*) in both ACRG and TCGA cohorts (Figs. 4J and S7I). On the contrary, positive correlations with mesenchymal markers such as N-Cadherin (*CDH2*), the cytoskeletal protein Vimentin (*VIM*), the matrix metallopeptidase 2 (*MMP2*), proteins belonging to the extracellular matrix (ECM) such as Fibronectin 1 (*FN1*) and Matrilin-3 (*MATN3*), or related to its composition such as the serpin family H member 1 (*SERPINH1*), were detected (Figs. 4J and S7I). Finally, positive correlations were found between the 5 genes with the expression of different EMT inducers including *ZEB1/2, SNAI1/2, TWIST1/2, PRRX1* and *STAT3* (Figs. 4J and S7I). Notably, the strongest correlations and with the highest number of markers of each group were detected for *ANKRD6* and *ITIH3.*

Since metastasis and EMT, as well as recurrence and therapy resistance, are attributed to cancer stem cells (CSCs) [31, 32], we studied the correlation of the 5 genes with regulators of CSCs. This analysis revealed that several stem cell markers were positively and significantly correlated with the 5 genes in both cohorts (Figs. 4K and S7J). As in the case of biological pathways and EMT markers/inducers, *ANKRD6* and *ITIH3* were the genes, specially the first one, presenting the strongest patterns of correlation and with a higher number of stem markers, exhibiting *ANKRD6* significant correlations with *SOX2, BMI1, KLF8, LINGO2, CXCR4, CD90* and *YAP1* (Figs. 4K and S7J). These results associate high levels of *ANKRD6* and *ITIH3* with the EMT process.

### *ANKRD6* is required for gastric cancer cell activity

**Fig. 5:**
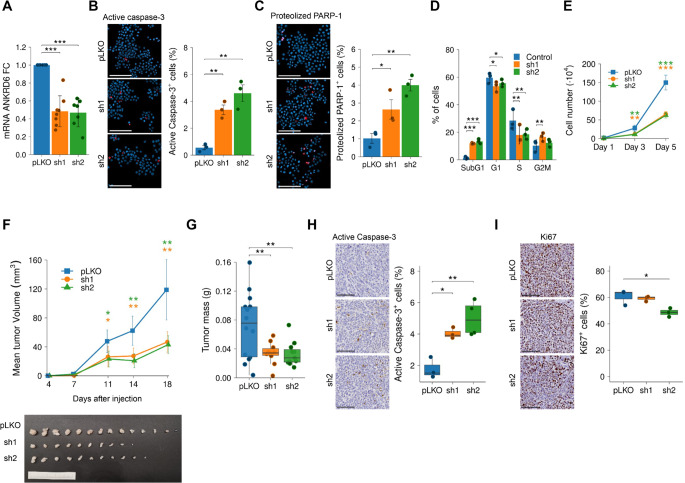
Role of *ANKRD6* in gastric cancer cell activity. **A** *ANKRD6* mRNA expression in MKN45 GC cell line lentivirally transduced with *pLKO* (control) or *ANKRD6* silencing (*sh1* and *sh2*) plasmids (n ≥ 3). Quantification and representative images of active Caspase-3-positive cells (**B**) and proteolyzed PARP-1-positive cells (**C**) in *pLKO* and *shANKRD6* MKN45 cells analyzed by immunofluorescence (n = 3). **D** Cell cycle distribution in *pLKO* and *shANKRD6* MKN45 cells assessed through the study of cell DNA content by flow cytometry (n = 3). **E** Growth curves representing the number of *pLKO* and *shANKRD6* MKN45 cells counted at days 1, 3 and 5 after seeding (n ≥ 3). **F** Volume at the indicated time points and image of subcutaneous tumors generated by *pLKO* (control) and *shANKRD6* MKN45 cells in immunodeficient FOXn1^nu (nu/nu)^ mice. **G** Mass of subcutaneous tumors represented in (F). Representative images and quantification of cells presenting active Caspase-3 (**H**) and Ki67 positive staining (**I**) assessed by IHC in subcutaneous tumors from (F). Scale bar: 100 µm

*ANKRD6* was the most robustly associated with poor outcome, recurrence and metastasis among the 5 genes. Moreover, its impact on GC cell activity remained unexplored. Therefore, we selected this gene in order to address its functional role in GC cells. For this, we silenced the expression of *ANKRD6* using 2 independent short hairpin RNAs in the metastatic GC cell line MKN45, which expresses high levels of *ANKRD6*, as observed in CCLE (Fig. S8B). Once we validated the significant inhibition of *ANKRD6* using both short hairpins (Fig. 5A), we analyzed the phenotype of the *ANKRD6*-silenced cells, detecting multiple cells de-attached in *sh1* and *sh2* conditions, indicative that they could undergo apoptosis. To confirm this idea, immunofluorescence of the apoptosis markers active Caspase-3 and proteolyzed PARP-1 were performed. Noteworthy, we detected an increase of around 5–10 fold in *ANKRD6-*silenced cells compared to control cells. In particular, the proportion of cells positive for active Caspase-3 was 3.37% ± 0.31 and 4.61% ± 0.54 in *sh1* and *sh2* cells compared to 0.56% ± 0.14 in controls (Fig. 5B). Similarly, proteolyzed PARP-1-positive cells were 2.64% ± 0.55 and 4.00% ± 0.32 in *ANKRD6*-silenced compared to 1.03% ± 0.27 in controls (Fig. 5C). Additionally, flow cytometry analysis unveiled a significant increase in the proportion of cells in the subG1 phase in *ANKRD6*-silenced cells (*sh1*; 11.87% ± 0.25; *sh2*: 13.39% ± 0.99) respect to control cells (0.77% ± 0.45) (Fig. 5D). Moreover, we observed an increase in the percentage of cells in the G2M phase, whilst the proportion of cells decreased in G1 and S phases (Fig. 5D), suggesting cell cycle arrest. To test the effect on cell proliferation, we performed cell count experiments, which revealed a significant and progressive reduction in the number of cells over time in both *sh* conditions compared to control cells (Fig. 5E). Concretely, at day 5, the number of *sh1* and *sh2* cells was reduced by ~60% (Fig. 5E).

Then, we moved to the in vivo setting and established subcutaneous xenografts of control and *ANKRD6*-silenced cells in immunodeficient mice. *ANKRD6*-silenced cells displayed a significant reduction in tumor growth with respect to control cells (Fig. 5F). The decrease in tumor volume was 67.2% in *sh1* tumors and 66.8% in *sh2* at day 18. Consistent with this, the tumor weight was significantly lower in those derived from *ANKRD6*-silenced cells (Fig. 5G). Furthermore, we explored Caspase-3 and the proliferation marker Ki67 in these tumors by IHC, finding a 2–3 fold increase in the number of active Caspase-3 positive cells (Fig. 5H), and a decrease in the proportion of Ki67-positive cells (Fig. 5I) in *sh* conditions, which confirmed in vivo the requirement of *ANKRD6* for tumor cell survival and proliferation. Overall, these results demonstrate that *ANKRD6* is required for GC metastatic cell activity and malignancy.

### *ANKRD6* is required for GC metastatic traits

**Fig. 6:**
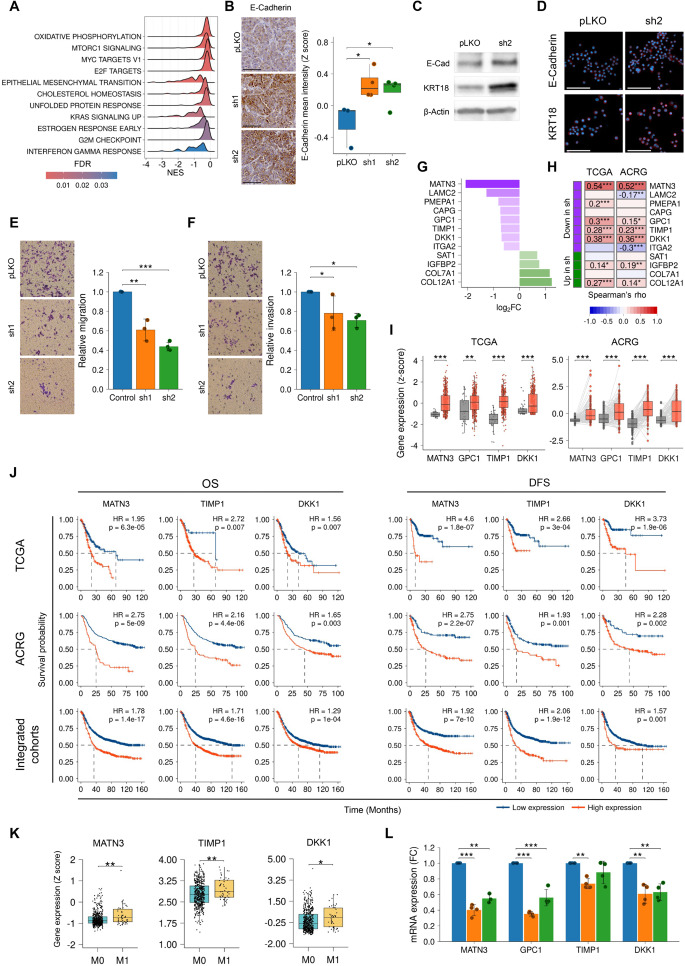
Pro-metastatic role of *ANKRD6* in gastric cancer. **A** GSEA ridge plots depicting the enrichment of signal pathways altered in *shANKRD6* cells respect to *pLKO* cells. **B** Representative image and quantification of E-Cadherin determined by IHC in subcutaneous tumors derived from *pLKO* and *shANKRD6* MKN45 cells. Scale bar: 100 µm. Representative western blot (**C**) and immunofluorescence (**D**) of E-Cadherin and KRT18 in *pLKO* and *sh2 ANKRD6* MKN45 cells. Scale bar: 100 µm. Representative images and relative migration (**E**) and invasion (**F**) of *ANKRD6*-silenced cells respect to control cells determined by transwell assays (n ≥ 3). **G** Genes belonging to the Epithelial mesenchymal transition (EMT) hallmark gene set that are differentially expressed in *shANKRD6* cells respect to *pLKO* cells. Genes down-regulated are represented in purple and up-regulated in green. **H** Correlation in TCGA and ACRG cohorts between the expression of *ANKRD6* and the DEGs from the EMT hallmark. **I** Expression of *MATN3, GPC1, TIMP1* and *DKK1* in normal gastric tissue (grey) versus the corresponding paired GC tissue (red) in TCGA (N_tumor_ = 32, N_normal_ = 375) and ACRG (N_tumor_ = 98, N_normal_ = 98). **J** Kaplan-Meier plots of OS (left) and DFS (right) according to the expression of *MATN3, TIMP1* and *DKK1* in TCGA, ACRG and the integrated cohort. **K** Expression of *MATN3, TIMP1* and *DKK1* according to M stage in the integrated cohort (N_M0_ = 513, N_M1_ = 45). **L** mRNA expression of *MATN3, GPC1, TIMP1* and *DKK1* determined by qPCR in *shANKRD6* respect to *pLKO* cells (n ≥ 3) (Colour figure online)

Then, to unravel the molecular mechanisms associated to the activity of *ANKRD6*, we performed RNA sequencing in control and *ANKRD6*-silenced cells. 450 genes were differentially expressed with respect to control cells, with a fold-change cut-off of >1.5 and *p*-adjusted values <0.05. Among them, 198 genes were up-regulated and 252 down-regulated. The gene set enrichment analysis (GSEA) identified statistically significant downregulation of 11 pathways in *ANKRD6-*silenced cells (Fig. 6A). Among them, the hallmark of EMT was identified, as well as other hallmarks such as G2M checkpoint or KRAS signaling up, which is in agreement with the results obtained above. Consequently, we studied functional and molecular events linked to EMT and metastasis in *ANKRD6*-silenced cells. First, we detected an increased expression of the epithelial markers E-Cadherin and Keratin-18 (KRT18) in *ANKRD6*-silenced cells using IHC, western blot and immunofluorescence techniques in vivo and in vitro (Fig. 6B–D). In line with these findings at cellular level, the expression of *ANKRD6* inversely correlated with both epithelial markers in the GC samples from the ACRG and TCGA patients (Figs. 4J and S7I). Moreover, the capacity of GC cells for migration and invasion was significantly impaired by *ANKRD6* silencing (Fig. 6E, F). Thus, the migration capacity of *ANKRD6*-silenced cells compared to controls represented the 60.8 ± 11.2% and the 43.7 ± 4.3% for *sh1* and *sh2* cells, respectively (Fig. 6E). Similarly, the invasive potential of *sh* cells was reduced in around 30% (Fig. 6F).

We observed that within the hallmark of EMT, 8 genes were significantly down-regulated and 4 were up-regulated in *ANKRD6*-silenced cells (Fig. 6G). The down-regulated genes were *PMEPA1*, a native regulator of TGF-beta; *DKK1*, an antagonist of the canonical Wnt signaling pathway; *CAPG*, which is involved in actin dynamics; and genes coding components of the ECM (*MATN3* and *LAMC2*) or ECM-interacting proteins (*ITGA2, GPC1* and *TIMP1*) (Fig. 6G). We moved back to the patients and found that *MATN3, GPC1, TIMP1* and *DKK1* were positively and significantly correlated with the expression of *ANKRD6* in the GC patients from both the TCGA and the ACRG cohorts (Fig. 6H). Additionally, their expression was higher in GC tissue respect to normal gastric tissue in both cohorts (Fig. 6I), whereas high expression of *MATN3, TIMP1* and *DKK1* was significantly associated with reduced OS and DFS in GC patients from the TCGA, ACRG and the additional cohorts used in this study (Fig. 6J), their expression being also significantly elevated in the samples of those patients presenting metastasis (Fig. 6K). Finally, we detected that the expression of the four genes was significantly downregulated in *shANKRD6* cells validating the in silico results (Fig. 6L). These findings, overall, reveal a role of *ANKRD6* in GC progression, metastasis and EMT.

## Discussion

Our work identified the top-genes most significantly associated with reduced, both OS and DFS of GC patients from the TCGA. These genes are *ANKRD6, ITIH3, SORCS3, NPY1R* and *CCDC178*, which form a signature associated to adverse clinic-pathological features such as advanced tumor stage, poor prognosis and disease recurrence in GC. Among them, *ANKRD6* and *ITIH3* are the most significant genes within the signature. *ANKRD6*, also called Diversin, belongs to the ankyrin repeat domain protein family and is the homolog of the Diego protein of *Drosophila* [33], whilst *ITIH3* encodes a protein that binds hyaluronic acid and stabilizes the ECM [34]. Very little is known regarding their expression and activity in GC and those findings complement our results. Thus, a recent work proposed a cuproptosis-related prognostic 3-gene signature including *ANKRD6* [35], whereas in relation to ITIH3, higher levels were detected in the plasma of GC patients respect to healthy individuals [36, 37], and it was part of a 8-gene signature associated with poor outcome [58]. Additionally, a few works connected their expression with outcome in additional types of cancer. In this sense, *ANKRD6* is overexpressed in breast cancer, wherein its high level is associated with advanced TNM stages and nodal invasion [38]. In gliomas, the positive expression of *ANKRD6* is associated with advanced tumor grade [39], whereas in non-small cell lung cancer (NSCLC) and colorectal cancer (CRC) is correlated with poor prognosis, advanced stages and poor differentiation [40–42]. Referring to *ITIH3*, higher levels of ITIH3 in the plasma were reported in lung [43], pancreatic [44], and breast cancer [45], whereas opposite findings have been detected in colorectal cancer [46, 47]. In tumor biopsies, *ITIH3* mRNA is reduced in tumor versus normal tissue in breast, uterus, colon, ovary, lung, rectum, and prostate cancer [48, 49], and low protein levels were significantly associated with poor prognosis and platinum resistance in ovarian cancer [49]. These results suggest that the effect of *ITIH3* in cancer is context-dependent, acting as a tumor suppressor in some cancer types, whilst it may exhibit an oncogenic role in others including GC. Indeed, our work has shown that high *ITIH3* expression is associated with reduced survival also in adrenocortical carcinoma, colon adenocarcinoma, kidney renal clear cell carcinoma, kidney renal papillary cell carcinoma and mesothelioma. Moreover, our results confirm that *ANKRD6* is commonly overexpressed in cancer where it is associated to poor outcome and malignancy.

Regarding the other 3 genes of the signature little is known about their expression and role in cancer. *NPY1R* encodes a G protein-coupled receptor that mediates the function of the neurotransmitter neuropeptide Y (NPY), and the peptide YY (PYY), which is a gastrointestinal hormone that controls appetite [50]. *CCDC178* encodes a protein located in the ciliary basal body, whilst *SORCS3* encodes a receptor transmembrane protein of the vacuolar protein sorting 10 family, which acts as a postsynaptic modulator of synaptic depression [51]. Specifically in GC, *NPY1R* is more highly expressed in the subgroup of gastric tumors presenting *MUC16* mutations, and, in agreement with our results, the high expression of *NPY1R* was associated with dismal prognosis in the GC patients from the TCGA and ACRG cohorts [52]. In the case of *SORCS3,* it has been detected its methylation [53] and this epigenetic modification has been associated with the progression of gastric precancerous lesions [54]. In the case of *CCDC178,* an study that performed whole genome sequencing in samples from a GC patient, identified in the primary GC tumor a somatic nonsynonymous single nucleotide variation affecting *CCDC178* [55], and another study identified in GC mutations (mainly missense mutations and frame shift deletions) in *CCDC178* that were significantly associated with poor survival [56].

We report the potential of the 2-gene signature composed by *ANKRD6* and *ITIH3,* which predicts survival regardless of the tumor stage and the patients’ age, exhibiting HRs higher than those of the 5 genes individually, and whose score is higher in patients presenting recurrent disease. Additionally, we have also identified that both are involved in metastasis and EMT process. These results have been obtained taking advantage of public databases and bioinformatics from TCGA and ACRG cohorts, but also generating a new analysis pipeline allowing the study of almost 2000 CG patients derived from 9 independent cohorts. This pipeline represents a useful tool to analyze recurrence, therapy resistance and metastasis. Since no previous studies have explored the role of the identified genes in GC, we went deeper into the analysis of the relevance of the novel signature and characterized the role of *ANKRD6* in GC through functional studies in vitro and in vivo, and transcriptomic analysis to unravel downstream targets. These experiments complemented the results obtained in the bioinformatic analyses and unraveled that *ANKRD6* confers pro-metastatic and tumorigenic properties to GC cells, preventing apoptosis, promoting GC cell migration and invasion, fostering tumor growth, and regulating EMT related pathways but also hallmarks such as G2M checkpoint or KRAS signaling. In this sense, *ANKRD6* has been previously linked to WNT signaling, a critical pathway in GC, where it promotes the non-canonical Wnt/Planar cell polarity pathway [57].

Overall, our work identifies a novel signature of 5 genes associated with adverse outcomes in GC, driven specially by 2 of them, *ANKRD6* and *ITIH3,* and reveals for the first time the role of *ANKRD6* in metastasis and EMT process, pointing out their potential as a prognostic signature and molecular target in cancer.

### Electronic supplementary material

Below is the link to the electronic supplementary material.

Supplementary Material 1

## Data Availability

RNAseq data are deposited in NCBI’s Gene Expression Omnibus (GSE238103).
