# Supplementary figures and images for: Identification of a novel gene signature related to prognosis and metastasis in gastric cancer
Source: Cell Oncol (Dordr). 2024 Mar 13;47(4):1355–73. doi: 10.1007/s13402-024-00932-y (PMC11322236; doi:10.1007/s13402-024-00932-y)

A

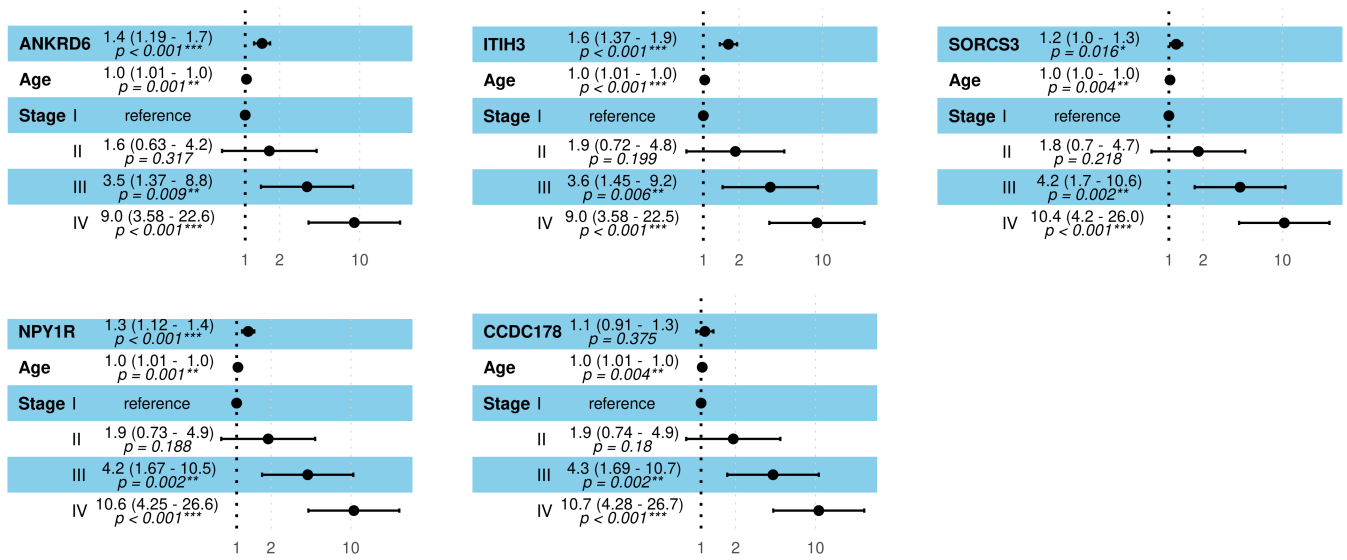

B

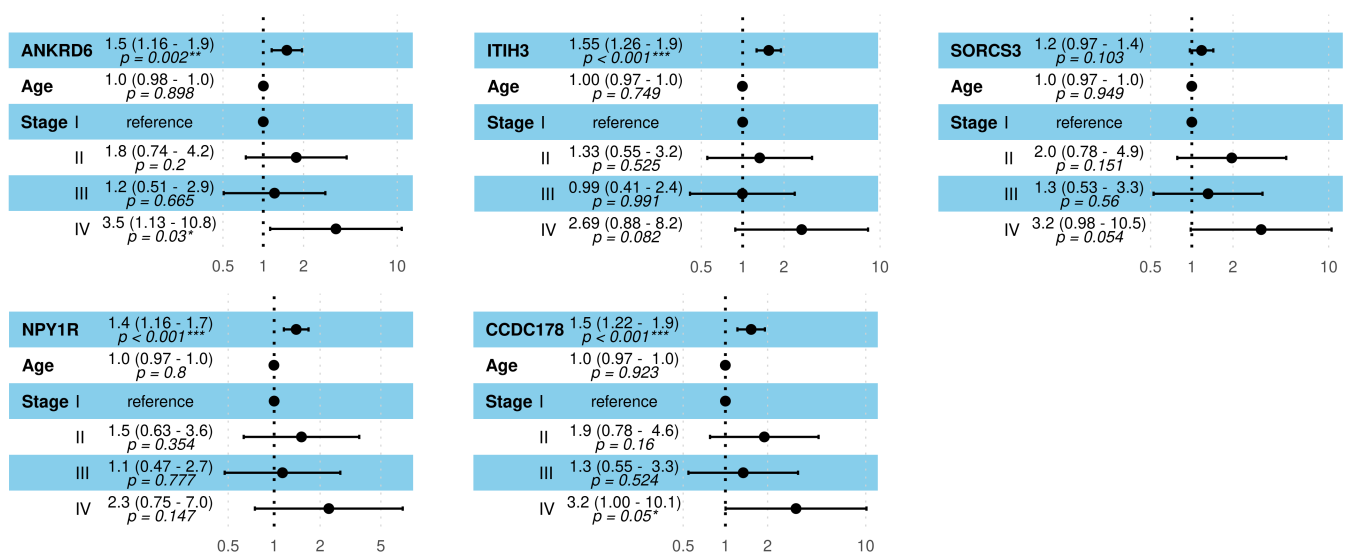

C

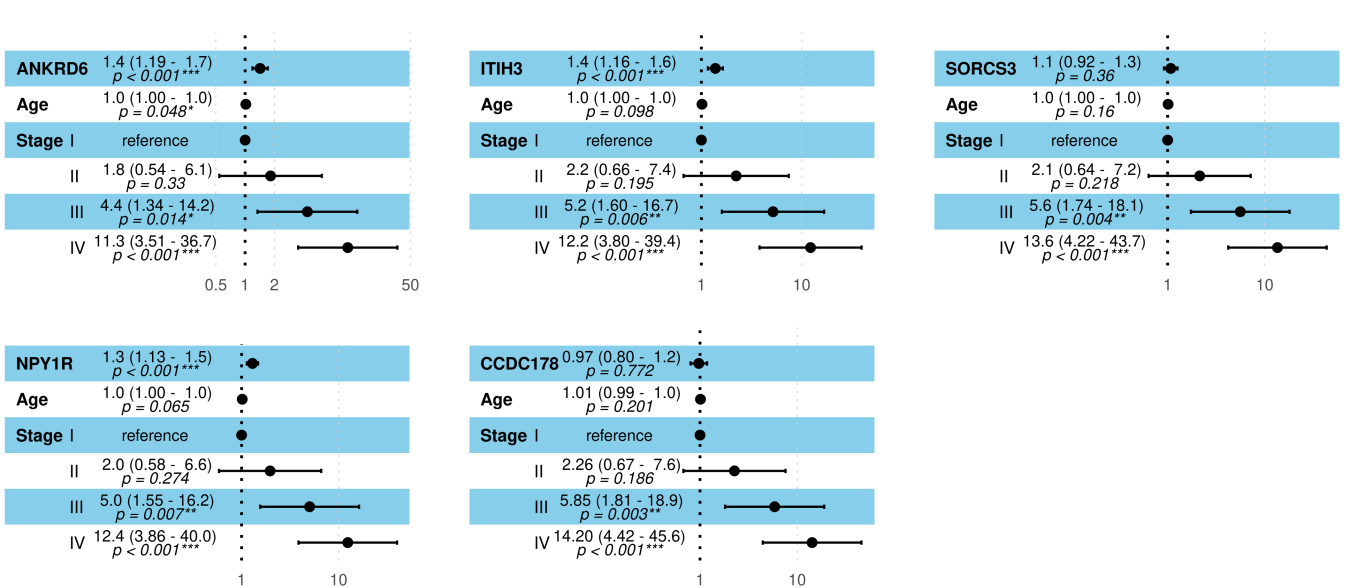

D

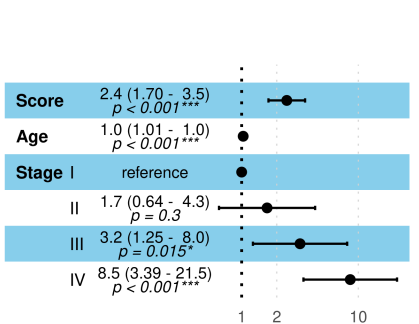

E

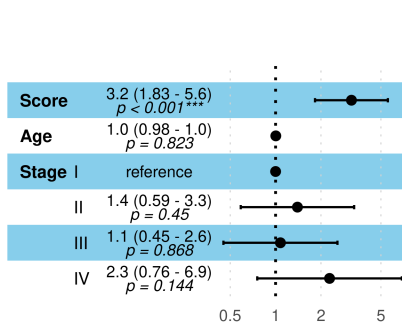

F

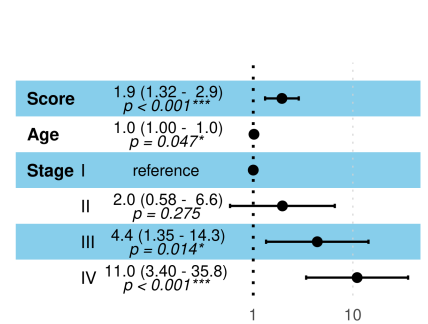

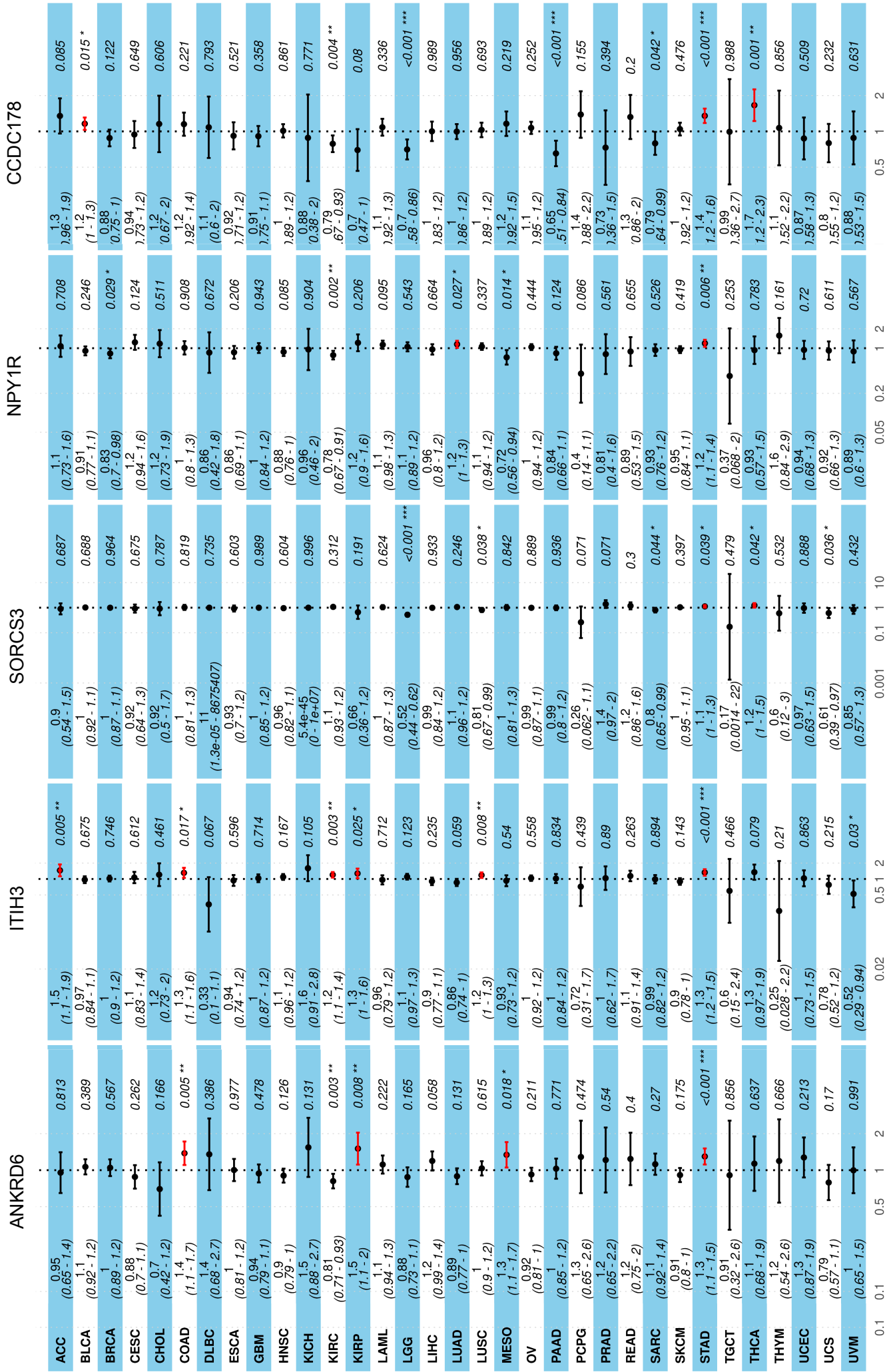

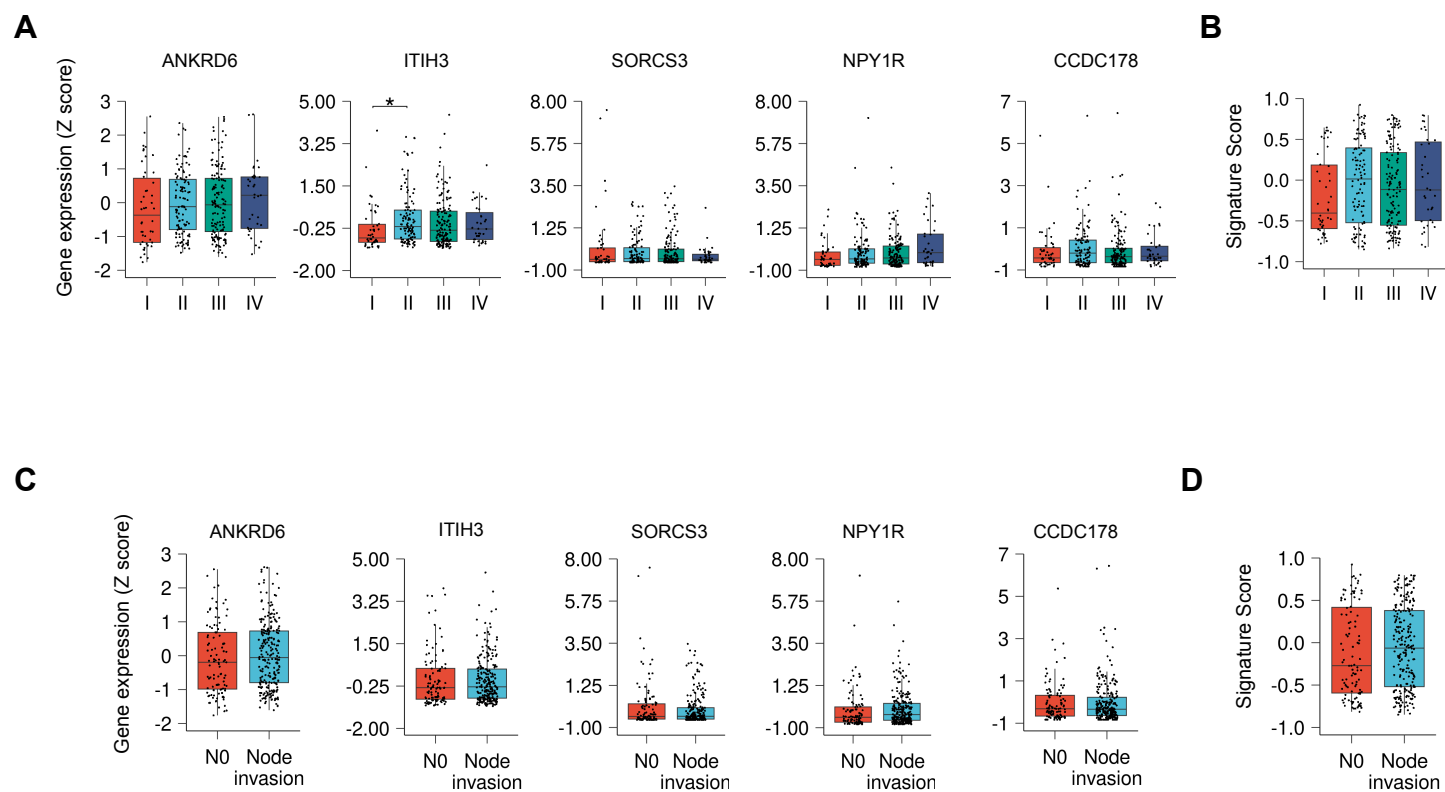

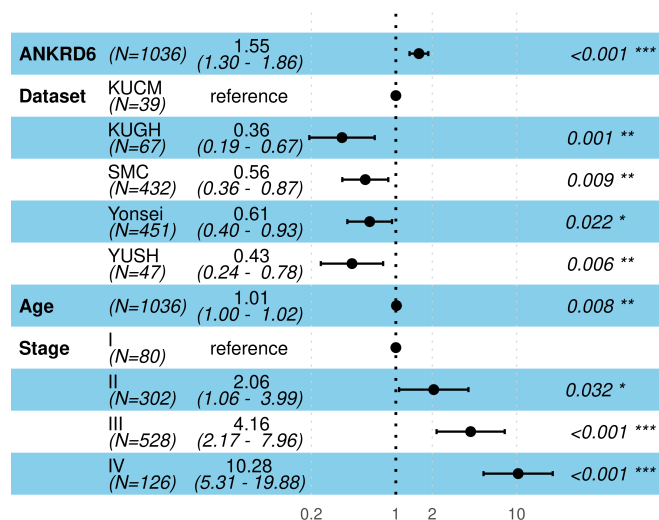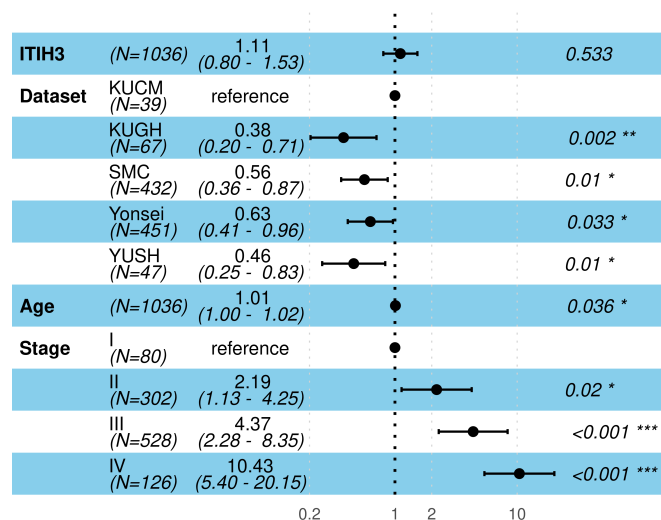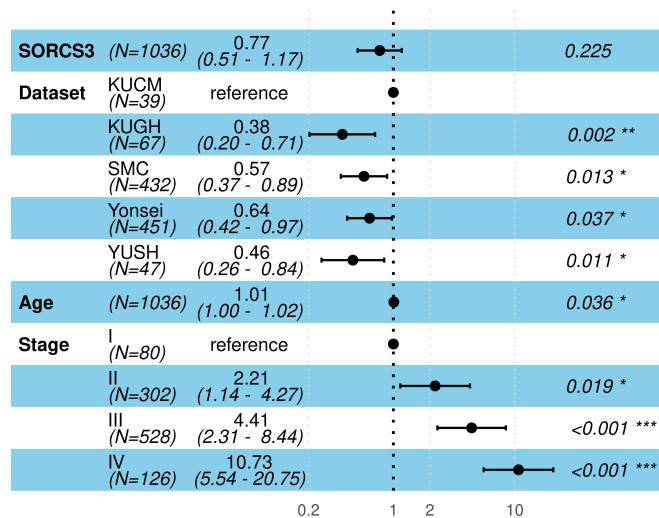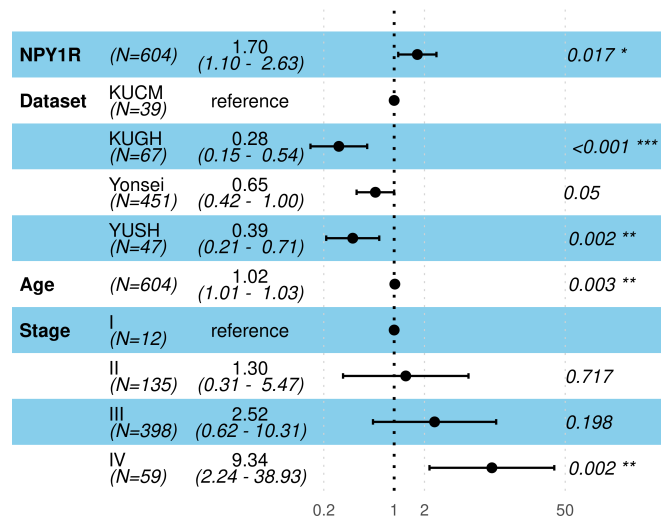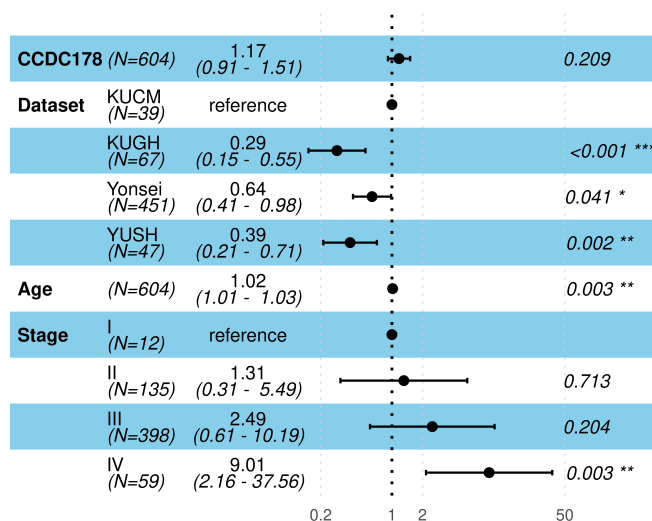

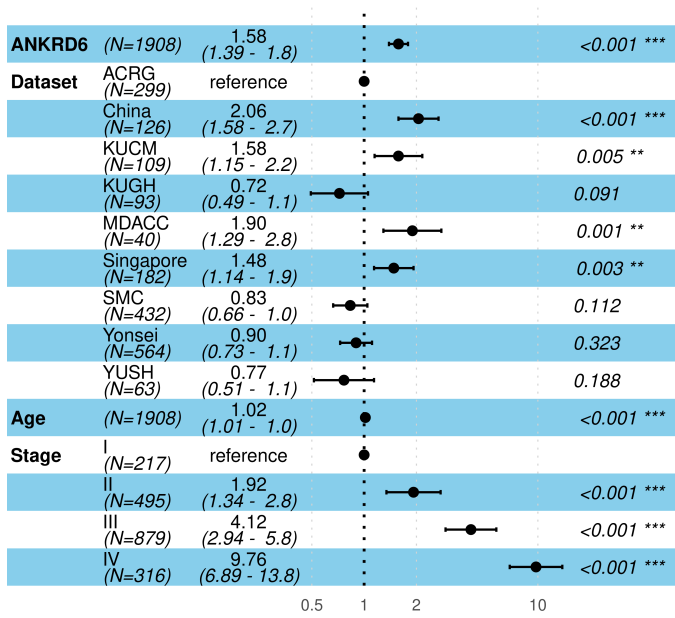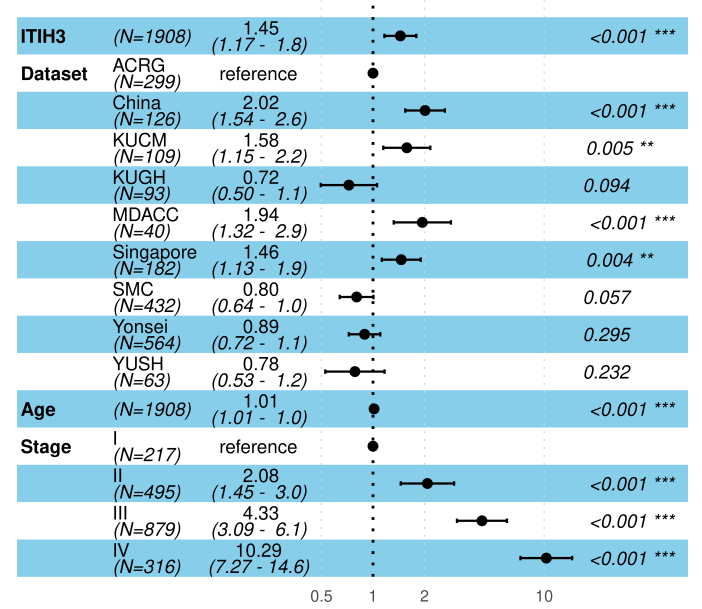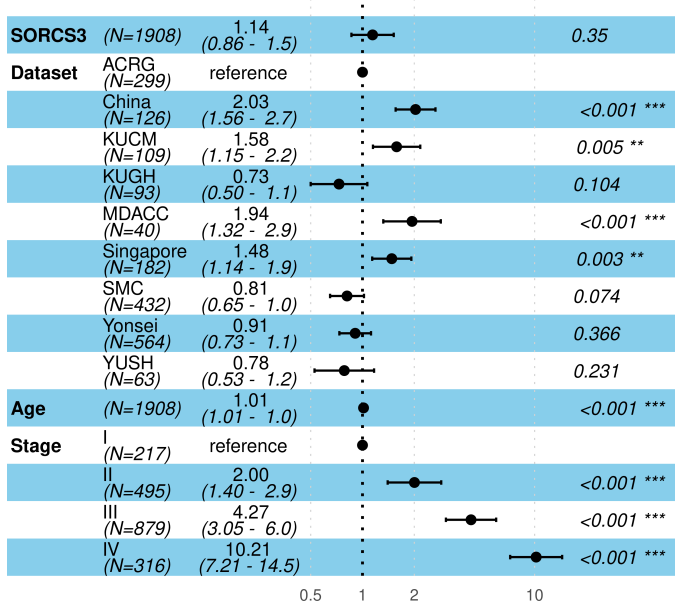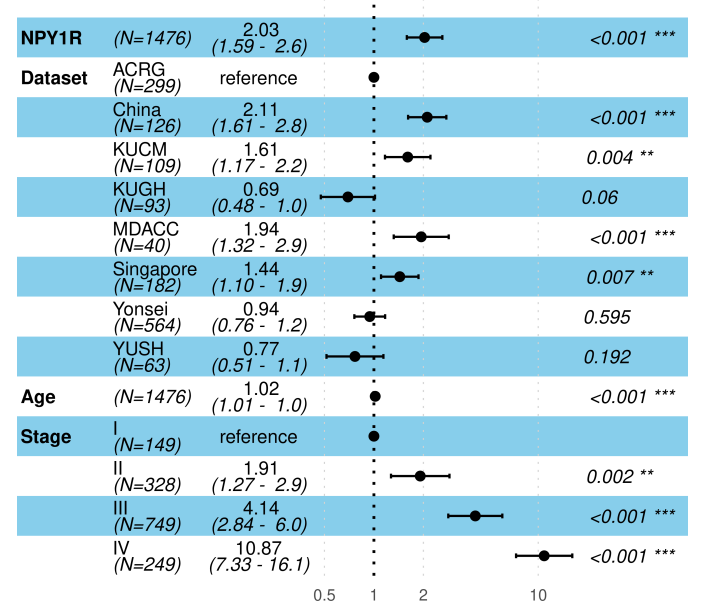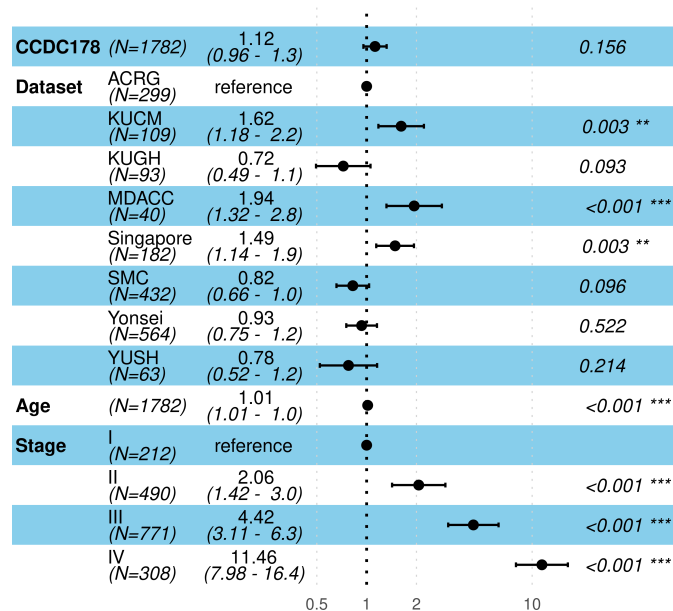

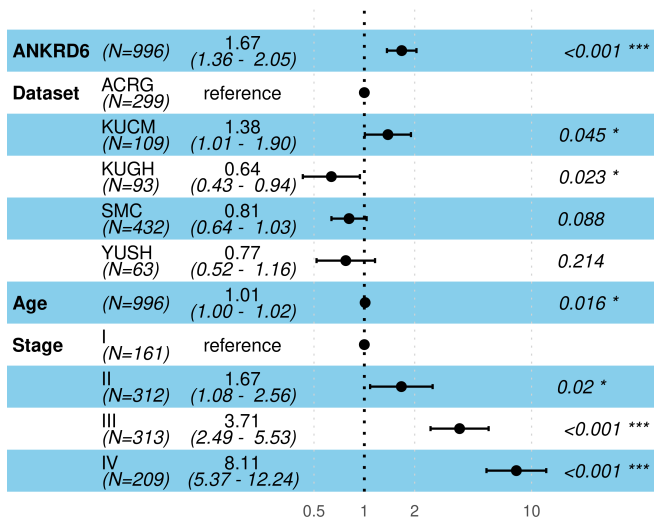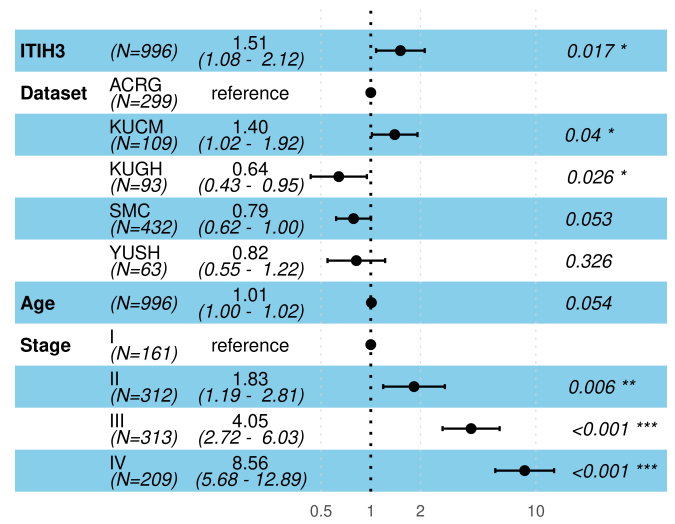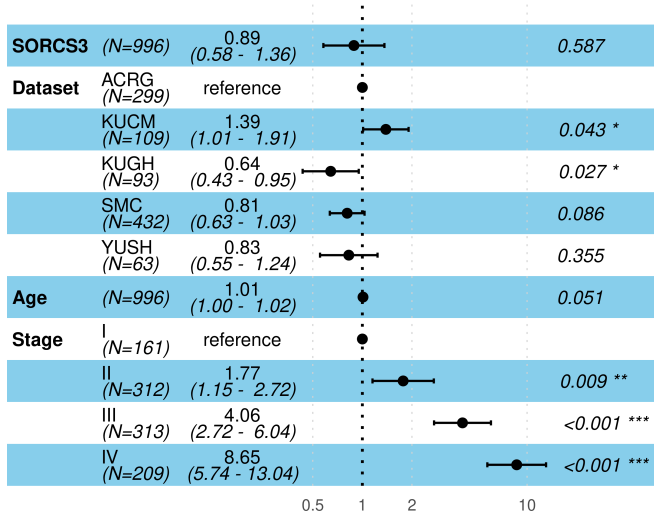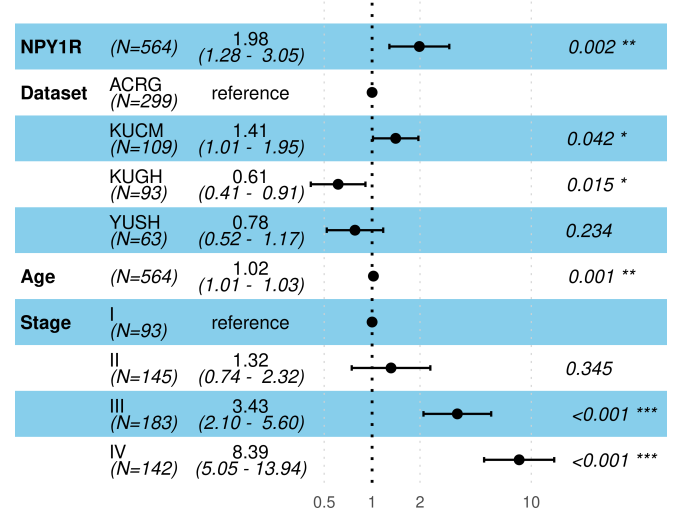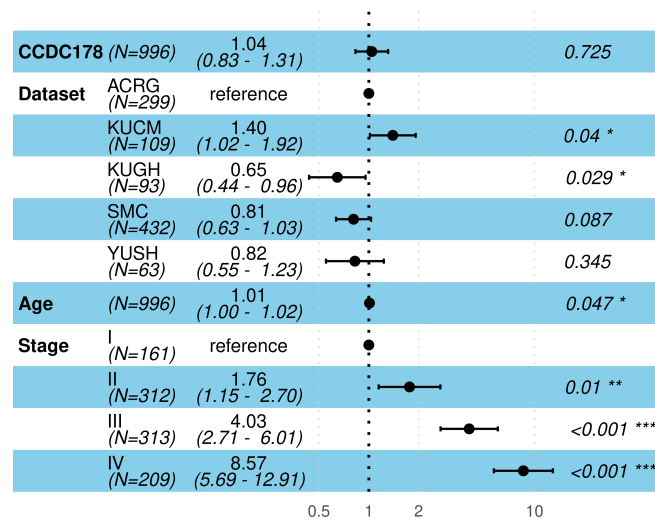

Fig. S7

A

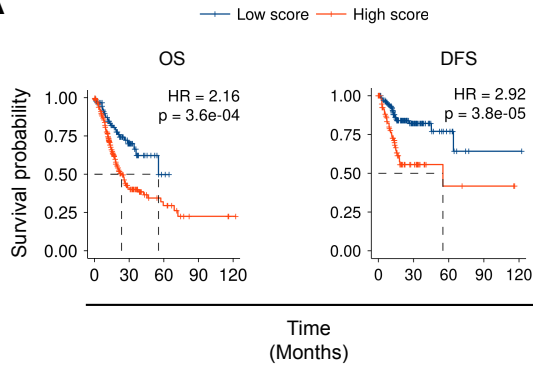

B

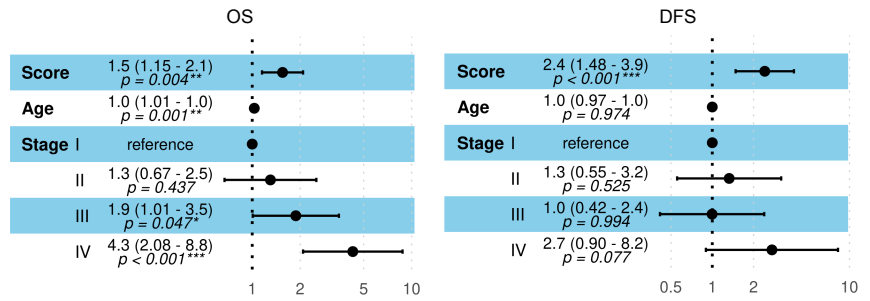

C

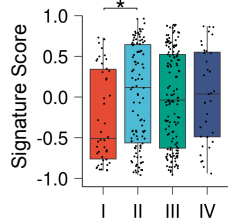

D

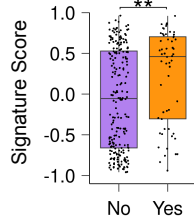

E

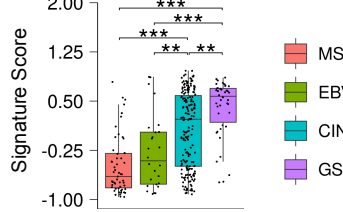

F

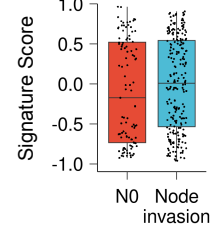

G

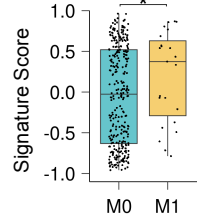

H

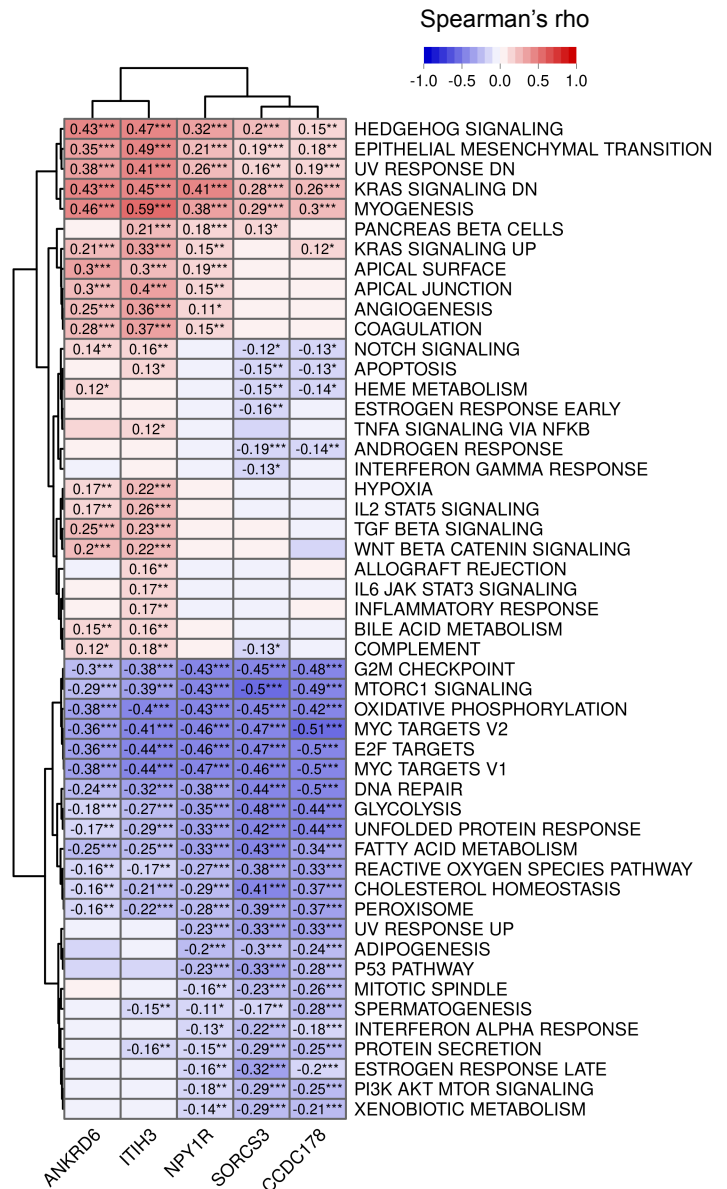

I

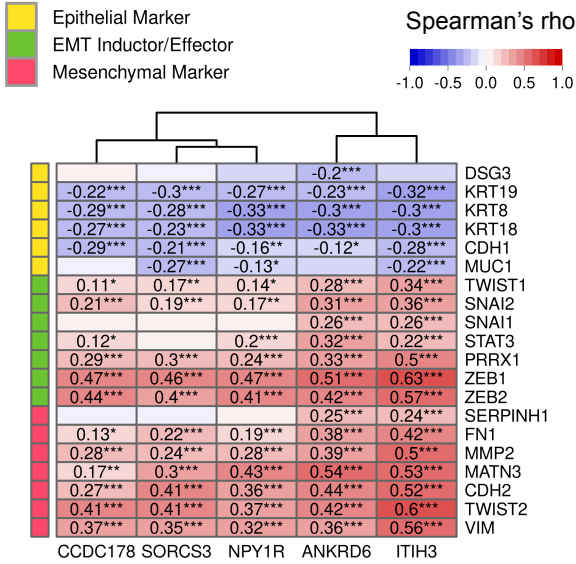

J

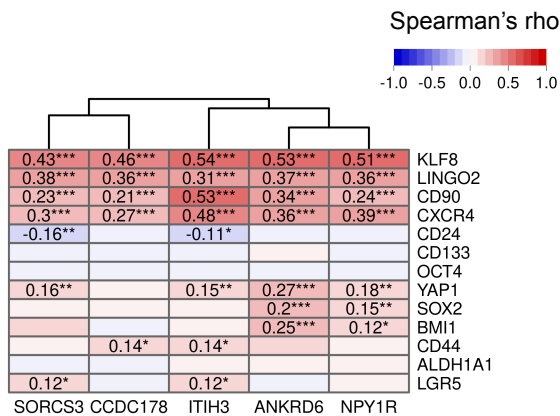

**A**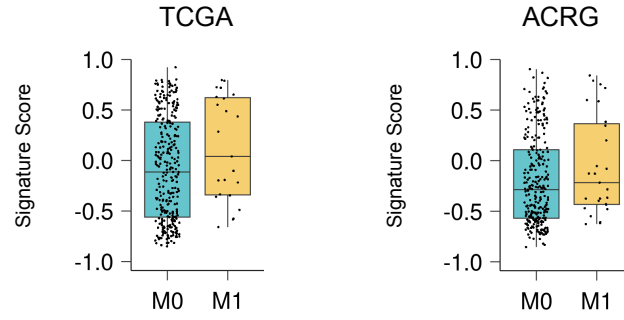**B**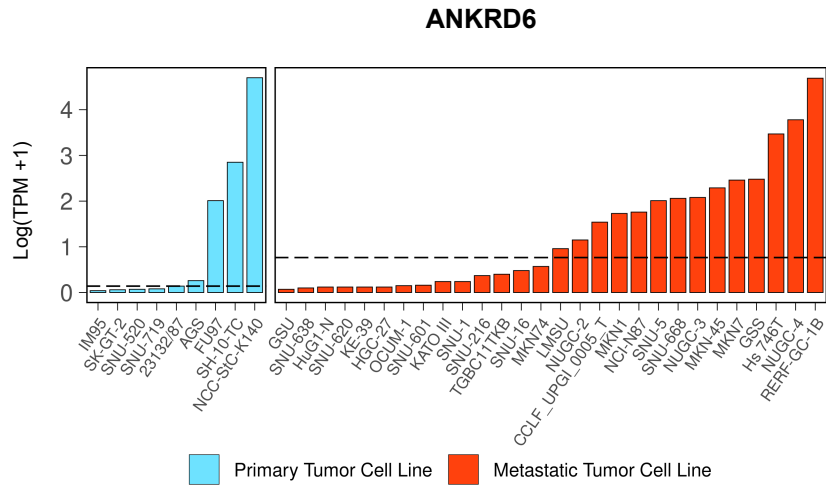**C**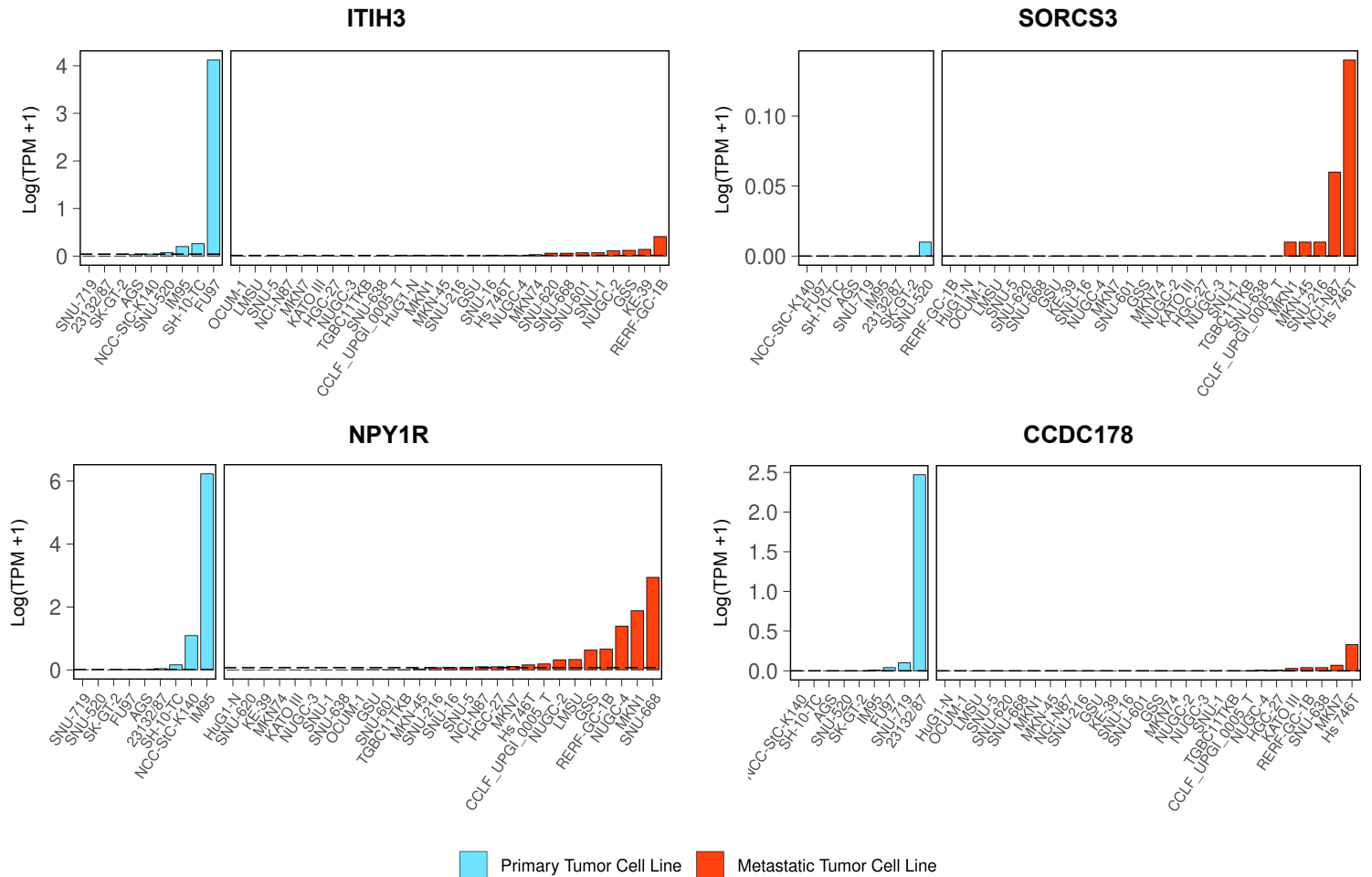

Supplement: Supplementary file 1 — Supplementary Material 1 [file 13402_2024_932_MOESM1_ESM.pdf]
